# Biochemical and genomic characterization of last-resort antibiotic resistance and oxidant tolerance in environmental *E. coli*

**DOI:** 10.3389/ftox.2026.1822843

**Published:** 2026-06-03

**Authors:** Asmaa Al-Mesaifri, Reem Ali, Abdallah Alhaj Sulaiman, Mustapha Aouida, Nisar Ahmed, Dindial Ramotar

**Affiliations:** 1 Division of Genomics and Precision Medicine, College of Health and Life Sciences, Hamad Bin Khalifa University, Doha, Qatar; 2 Division of Biological and Biomedical Sciences, College of Health and Life Sciences, Hamad Bin Khalifa University, Doha, Qatar; 3 Qatar Biomedical Research Institute, Hamad Bin Khalifa University, Doha, Qatar; 4 Department of Biotechnology, Faculty of Life Sciences & Informatics, Balochistan University of Information Technology, Engineering and Management Sciences, Quetta, Pakistan

**Keywords:** antibiotics, DNA damage and repair, drug resistance, *Escherichia coli*, mcr-1, oxidative stress, UVC

## Abstract

Colistin is currently reserved as a last-resort antibiotic for multidrug-resistant Gram-negative bacteria such as pan-resistant *Escherichia coli*. Colistin-resistant *E. coli* strains have been detected in wastewater, clinical, and agricultural sites, and this resistance is mediated by the plasmid-borne *mcr-1* gene, which can spread into other sectors, including the food chain. Herein, we isolated several *E. coli* strains from mixed wastewater that showed varying resistance to colistin with IC_50_ values ranging from 6.5 to 27.3 μg/ml as compared to 0.5 μg/mL for the wild-type strain. The strains all contained the *mcr-1* gene, and there is no gene duplication to account for the increased resistance to colistin. In addition, the strains displayed resistance to several other antibiotics belonging to different classes, including ciprofloxacin, sulfamethoxazole, and tetracycline, while maintaining wild-type sensitivity to other antibiotics such as tazocin and meropenem. Interestingly, some of the strains showed resistance to the powerful DNA-damaging agents, ultraviolet radiation and hydrogen peroxide, used for eradicating bacteria. Measurement of catalase activity, which decomposes hydrogen peroxide, was not significantly elevated, excluding the possibility that catalase is involved in the resistance of the strains to H_2_O_2_. However, we found that H_2_O_2_ treatment caused the accumulation of fragmented chromosomal DNA in the wild-type, but not in one of the representative H_2_O_2_-resistant strains. Analysis of whole genome sequencing data revealed that the H_2_O_2_-resistant strains harbour a high level of missense mutations in several genes, including DNA repair genes that could encode variant proteins with elevated capacity to repair damaged DNA.

## Introduction

Colistin, also called polymyxin E, was first discovered in 1947 by the Japanese scientist Yoshimura Koyama and his colleagues (Ito and Koyama, 1972). It was isolated from a soil bacterium called *Paenibacillus polymyxa* and the producing strain was referred to as *B. polymyxa* var. *colistinus*, from which the name “colistin” was derived. During the period 1970–1990, colistin was developed and introduced clinically as an antibiotic effective against Gram-negative bacteria (Gugliandolo et al., 2017). At around the same time, colistin was used as an additive to the feed for poultry and animal husbandry to prevent bacterial infections and promote the growth of livestock (Li et al., 2019; Mazutti et al., 2016). However, in the 1970s, it was discovered that colistin caused kidney damage and neurotoxicity (Spapen et al., 2011), and as a result, its use in clinics was greatly diminished, while colistin usage continued in livestock farming. By 2000, and despite colistin side effects, it re-emerged as a last-resort antibiotic against multidrug-resistant organisms such as *Acinetobacter baumannii*, *Klebsiella pneumoniae,* and *Pseudomonas aeruginosa*, as these bacteria began developing resistance to nearly all major classes of antibiotics, including β-lactams (e.g., carbapenems), fluoroquinolones, and aminoglycosides. As such, it became a concerted effort in 2006, necessitated by several organizations, including infectious disease experts and hospital networks worldwide, for the WHO and CDC to implement colistin as a salvage therapy for treating severe bacterial infections (Ahmadpour et al., 2022; Vlad et al., 2025; Li et al., 2006).

The bacterial outer membrane is composed of lipopolysaccharides (LPS) with negatively charged phosphate groups that are tightly cross-bridged by divalent cations, mainly Mg^2+^ and Ca^2+^. These divalent ions act like molecular “glue”, neutralizing charge repulsion by LPS and stabilizing the outer membrane structure and protecting the bacteria. Replenishing damaged LPS requires a significant fitness cost to the bacteria. Colistin is a cationic lipopeptide with positively charged diaminobutyric acid residues and acts by forming a strong electrostatic interaction with the negatively charged LPS. This interaction competitively displaces the Mg^2+^ and Ca^2+^ from LPS, promoting electrostatic repulsion between the LPS molecules and loss of structural integrity of the membrane (Velkov et al., 2010; Velkov et al., 2013; Andrade et al., 2020). In addition, the lipophilic fatty acyl tail of colistin can insert into the membrane, causing it to become permeable due to pore formation, leading to the leakage of intracellular contents, loss of membrane potential, rapid cell lysis, and death (Mondal et al., 2024). Thus, colistin was reintroduced because in many cases it was the only drug that remained effective, but it accelerated the emergence of resistance and is now a major concern in both clinical and environmental settings.

The discovery of the plasmid-borne mobilized colistin-resistant gene 1 (*mcr-1*) in 2015, and its first transmission and detection in a patient in the United States, prompted more vigorous experimentation to understand bacterial resistance to the drug (Kline et al., 2016). As such, following the discovery of the *mcr-1* gene, various countries started in 2016 to restrict the use of colistin in animal feed for promoting growth (Nagy et al., 2021), and by 2022, the European Union completely prohibited the use of colistin in livestock farming (Ahmed et al., 2025). The *mcr-1* gene encodes a small enzyme, and in the presence of Zn^2+^, transfers a phosphoethanolamine group onto the 4′-phosphate and sometimes 1′-phosphate position of the lipid A disaccharide. This reduces the net negative charge of the cell surface from −1.5 to approximately −0.5, creating the repulsive force to minimize interaction with the positively charged colistin (Velkov et al., 2010; Ma et al., 2016; Kline et al., 2016; Eltai et al., 2018). It is predicted that any increase in colistin resistance will be an enormous challenge to use the drug as a last-resort antibiotic to treat multidrug-resistant Gram-negative infections (Zeb et al., 2026). Consistent with this prediction, there have been reports regarding the extreme resistance of Gram-negative bacteria, such as *Escherichia coli*, towards colistin treatment (Al Momani et al., 2024), suggesting that there are likely other contributing factors besides the presence of the *mcr-1* gene. Since colistin at high concentrations can cause kidney toxicity (Spapen et al., 2011), it makes it difficult to apply existing therapeutic dosages and regimens to treat bacterial infections that are highly resistant to colistin (Kline et al., 2016). This underscores the need to identify and characterize additional genes from bacteria that can contribute to colistin resistance (Nagy et al., 2021; Alhababi et al., 2020). These genes can be added to the growing number of antimicrobial resistance genes that should be screened for antibiotic resistance to provide optimal benefits for patient treatment, as well as monitor and prevent the spread of evolving colistin-resistance strains (Janssen and van Schaik, 2021).

There is a paucity of data on the occurrence of variation in the resistance to colistin in various environmental sectors, such as crops, poultry farms, and wastewater from hospitals, municipalities, and industries (An et al., 2023). We have recently reported the partial characterization of three colistin-resistant *E. coli* strains isolated from a mixture of wastewater samples (Alhaj Sulaiman et al., 2025). The strains (DSR2, 3, and DNR3) carry the *mcr-1* gene on distinct plasmids (IncI2, IncX4, and IncHI2A) and show resistance to multiple antibiotics, while retaining sensitivity to some classes of antibiotics, such as carbapenems and cephalosporins (Alhaj Sulaiman et al., 2025). These strains also exhibited different adherence properties on surfaces and thus have the propensity to sustain environmental challenges and propagate. Wastewater is a complex milieu composed of various toxic inorganic and organic chemicals, as well as heavy metals, that can all act as mutagens (Kato and Kansha, 2024). It provides the surroundings that can cause bacteria to easily mutate and become resistant to antibiotics, escape wastewater treatment conditions, and adapt to harsh environmental conditions to promote horizontal gene transfer and the propagation of mutated genes for adaptive stress responses (Kato and Kansha, 2024).

In this study, we examined *E. coli* strains obtained from wastewater samples to determine whether there are differences in the responses to colistin. We further check whether the colistin-resistant *E. coli* strains would display cross-resistance to other classes of antibiotics, as well as to disinfectant agents used in wastewater treatment plants, such as the chemical oxidant hydrogen peroxide (H_2_O_2_) and ultraviolet light (UVC) irradiation that destroys viruses and non-pathogenic and pathogenic organisms before the treated water is discharged. We report the presence of *E. coli* strains with extreme resistance to colistin, which is unlikely to be explained by variation in the expression of the *mcr-1* gene. We provide evidence that some of the strains were highly resistant to H_2_O_2_ and displayed varying resistance to UVC. The resistance to H_2_O_2_ is unrelated to the level of catalase activity, which decomposes H_2_O_2_. Whole genome sequence analysis of the colistin-resistant strains uncovered genomic mutations within DNA repair genes that could explain the striking resistance to H_2_O_2_ and UVC, as compared to the wild-type strain.

## Materials and methods

### Strains and media

Wild-type *E. coli* K12 DH5α (Thermo Fisher Scientific, United States), Colistin (Abcam, Cambridge, United Kingdom), meropenem and tazocin (obtained from Hamad Medical Corporation pharmacy), buffered peptone water (Thermo Fisher Scientific, United States), Luria-Bertani broth (LB) miller’s (Invitrogen, United States), rapid *E. coli*’2 (Bio-Rad, United States), Mueller-Hinton (MH) powder media (Thermo Fisher, United States), agar powder (Formedium, United Kingdom), ultrapure glycerol (Invitrogen, United States), sodium m-arsenite (Sigma-Aldrich, Germany), Dulbecco’s Phosphate-Buffered Saline (DPBS) – Gibco (Thermo Fisher Scientific, United States), RNASE/DNASE free water–Gibco (Thermo Fisher Scientific, United States), Dimethyl Sulfoxide (DMSO) (Invitrogen, United States), 6% hydrogen peroxide solution (Mexo, Kohen fine chemicals and pharmaceutical, India), autoclaved Milli-Q water.

### *E. coli* strains isolation, storage, and culture conditions

A total of 15 bacterial *E. coli* strains were isolated from influent and effluent wastewater samples obtained from various regions in Qatar, including the central part and the municipal sewer of Education City. The strains were referred to as a mixed source from wastewater, and they were given the nomenclature AAS to reflect the author who initially characterized the strains as AAS-1, AAS-2, AAS-5, AAS-6, AAS-7, AAS-8, AAS-9, AAS-10, AAS-11, AAS-12, AAS-14, AAS-18, AAS-20, AAS-21, and AAS-22. The strains were stored as glycerol stocks at −80 °C, and when needed grown in LB broth at 37 °C for 18–24 h or on solid LB agar plates for subsequent experiments (Alhaj Sulaiman et al., 2025).

### Minimum inhibitor concentration of *E. coli* strains towards colistin

Colistin broth microdilution assay was prepared as previously described (Alhaj Sulaiman et al., 2025). Briefly, strains (AAS-2, AAS-6, AAS-7, AAS-14, AAS-18, AAS-20, AAS-21) were randomly selected and cultured in 5 mL LB overnight at 37 °C with shaking in slightly capped tubes. The next day, the OD_600_ was measured using a Bio-spectrophotometer basic (Eppendorf, Germany) and adjusted to OD_600_ of 0.05 in 2 mL of liquid MH broth for each strain. A volume of 100 µL of the diluted cells in MH broth was added to 96-well plates each containing 20 µL of serially diluted colistin to final concentrations ranging from 0.125 to 128 μg/mL. Control wells have no colistin. The 96-well plate was placed in a plate reader (TECAN SPARK multi-mode microplate reader, Tecan, Switzerland) with shaking at 37 °C and OD_600_ taken periodically to monitor growth.

### Detection of the *mcr-1* gene

The colistin-resistant strains were streaked for single colonies onto MH agar plates containing 4 μg/mL colistin, while the wild-type strain was streaked onto LB agar and the plates were incubated overnight at 37 °C. The following day, single colonies of the wild-type and the colistin-resistant strains were cultured in liquid LB until growth reached an OD_600_ of 1. The cultures were spun at 4,000 rpm for 4 min, and genomic DNA extraction was performed using the QIAamp UCP pathogen extraction kit (QIAGEN, United States) as per the supplier protocol. DNA concentration and purity were measured using a Nanodrop. The DNA was subjected to PCR analysis using the 2x Phusion polymerase (Thermo Fisher Scientific, United States). Each reaction consisted of 10 µL Phusion polymerase, 1 µg template DNA in 1–3 µL nuclease free water, 0.5 µL forward primer (10 µM), 0.5 µL reverse primer (10 µM), and brought to a final volume of 20 µLwith 6–9 µL of nuclease-free water). The samples were then placed in a thermal cycler (Applied Biosystems, United States) using the following cycle conditions: (denaturation 95 °C, annealing 56 °C, extension 72 °C). To amplify the *mcr-1* gene, the Forward Primer: 5′-CGG​TCA​GTC​CGT​TTG​TTC-3′ and the Reverse Primer: 5′-CTT​GGT​CGG​TCT​GTA​GGG-3′ were used, and to amplify the *16SrDNA* housekeeping gene, the Forward Primer: 5′-AAG​AAG​CTT​GCT​TCT​TTG​CTG​AC-3′ and the Reverse Primer: 5′-AGC​CCG​GGG​ATT​TCA​CAT​CTG​ACT​TA-3′ were used. These primers were designed to amplify fragments of the *mcr-1* and the *16SrDNA* genes of 320 bp and 544 bp, respectively. The PCR products were loaded onto 1.5% agarose gel stained with SYBR Safe (ThermoFisher Scientific, United States), 1 kb O’Gene Ruler (ThermoFisher Scientific, United States) was used as a marker. Band detection was performed using ChemiDoc™ MP Imaging System.

### Antibiotic susceptibility testing

Briefly, the wild-type and strains AAS-14, AAS-18, and AAS-21 were cultured overnight in LB media in an incubator shaker at 37 °C. After 24 h, cultures were adjusted to an OD of 0.05, and 100 µL of this diluted cells were evenly spread onto Mueller-Hinton Agar (MHA) plates and allow to air dry for 10 min in a biosafety chamber. Antibiotic containing discs (Gentamicin 30 μg, cephalexin 30 μg, meropenem 10 μg, amoxicillin-clavulanic acid 3 μg, ciprofloxacin 10 μg, trimethoprim-sulfamethoxazole 25 μg, tetracycline 10 μg, and erythromycin 15 µg) were placed onto the MHA plates using sterile forceps. The plates were left to incubate at 37 °C overnight and the zones of inhibition were measured.

### H_2_O_2_ and UVC spot test analyses

The assay was done according to our previously published protocol, which can be adapted for either bacteria or yeast (Leduc et al., 2003). Briefly, the strains were grown in LB with overnight shaking at 37 °C. After 24 h, OD_600_ was adjusted to 0.1. The strains were then serially diluted to 1:10, 1:100, 1:1,000 and 1:10,000. LB agar plates were prepared with different concentrations of H_2_O_2_ (0, 0.25, and 0.5 mM), and 4 µL of each serial dilution of the strains was spotted onto plates without and with H_2_O_2_. The spots were left to visibly dry before the plates were placed in an incubator at 37 °C for overnight growth. For UVC exposure, the serially diluted strains were spotted onto the LB agar plates. Once the spots were dried, the plates were exposed to different UVC doses as indicated in the figure legend.

### Gradient plate assay

The assay was done according to our previously described method (Ramotar et al., 1991). Briefly, gradient plates (10 × 10 cm) were placed on an inclined surface (at least 15°), and 30 mL of LB agar with either H_2_O_2_ (2.94 mM) or menadione (19.14 μM) was poured and left to solidify. The plates were then placed flat, and another 35 mL of LB agar (without drug) was poured to create a gradient of drug concentrations (Ramotar et al., 1991). After solidifying and drying, 500 μL LB agar was mixed with 500 μL Milli-Q water, and 10 μL of overnight culture grown in LB was added and briefly vortex. The mixture was poured on a microscope glass slide prewarmed at 55 °C. Imprinting the strains AAS-14, -18, −21, and wild-type on agar was done using the side of another glass slide. Plates were incubated for 18–24 h at 37 °C and imaged using ChemiDoc™ MP Imaging System.

### Catalase assay

The assay was performed using a spectrophotometer to monitor the decrease in A240 absorbance of H_2_O_2,_ and the activity was expressed as units per mg total extract as previously described (Beers and Sizer, 1952).

### Assessment of H_2_O_2_-induced DNA damage by agarose gel electrophoresis

Overnight cultures were subcultured in LB and allowed to grow with shaking for 2–3 h at 37 °C. The exponentially growing cultures were either untreated, treated with 50 mM H_2_O_2_ for 30 min, or treated with 50 mM H_2_O_2_ for 30 min, washed twice with sterile water, resuspended in fresh LB, and allowed to recover for 2 h in fresh media. Cultures were spun at 4,000 × g for 5 min at 4 °C, the supernatant was discarded, and the pellets were used to extract the chromosomal DNA using the QIAamp UCP pathogen extraction kit (QIAGEN, United States) and the manufacturer’s protocol. The DNA was quantified by nanodrop, and 1 µg of each sample was analyzed by 1.0% agarose gel that was stained with SYBR safe to assess the genomic DNA integrity.

### Whole genome sequencing (WGS) and variant analysis

Genomic DNA was extracted from bacterial strains using the NucleoSpin Tissue Kit (Macherey-Nagel, Germany) following the manufacturer’s instructions. Extracted DNA was quantified and assessed for purity prior to submission. Samples were submitted for external sequencing at Macrogen Inc. (Seoul, Korea), where library preparation was performed according to Macrogen’s standard Illumina sequencing protocol. Paired-end sequencing was conducted on an Illumina HiSeq platform (sample identifier: AAS-14), and raw reads were quality-trimmed and assembled into FASTA format by the sequencing provider. Prior to downstream analysis, all sequencing data underwent quality control assessment to confirm sufficient genome coverage and read quality for reliable genomic characterization.

Raw paired-end sequencing reads were processed through a standard bacterial variant-calling workflow. Cleaned reads were aligned to the *Escherichia coli* reference genome NZ_CP014225.1 (4,659,625 bp), retrieved from the NCBI database, using the Burrows-Wheeler Aligner with the maximal exact matches algorithm (BWA-MEM, v0.7.17) with 30 threads. Split-read hits were marked as secondary alignments using the -M flag to ensure compatibility with downstream Picard-based tools and read group information was assigned at the alignment stage (ID: HWI; SM: LFE7; LB: HiSeq). Alignments were coordinate-sorted, indexed, and processed with Sambamba (v1.0) to mark and remove PCR and optical duplicates arising from library amplification. Variant calling was performed using SAMtools mpileup (v1.9) with a maximum per-site depth of 10,000 reads, piped directly into BCFtools call (v1.9) using the multiallelic calling model (-m) with variant-only output (-v). The resulting variant calls were filtered using BCFtools filter (v1.9) to retain high-confidence variants with a Phred-scaled quality score above 30 (QUAL > 30) and a minimum of five supporting reads on both strands for either the reference or alternate allele, as determined by the DP4 field. In addition, single-nucleotide polymorphisms (SNPs) located within 3 bp of an insertion/deletion (SnpGap filter) and insertions/deletions located within 10 bp of another insertion/deletion (IndelGap filter) were flagged to reduce false-positive calls in complex regions. Functional effects of the filtered variants, including missense, synonymous, upstream gene variants, and other predicted consequences, were annotated using SnpEff (v4.3t) against the NZ_CP014225.1 reference annotation, producing effect predictions, gene identifiers, impact classifications, and HGVS notation, as well as the semicolon-delimited annotation strings used in downstream analyses.

All downstream variant-level analyses for the experimental evolution line LFE7 were conducted in R (version 4.3.1; R Core Team, 2023) using a standardized custom analysis pipeline. Annotated variant data were imported from Excel files using the *readxl* package (v1.4.3), and subsequent data manipulation, filtering, and summarization were performed using the *tidyverse* suite of packages (v2.0.0), including *dplyr* (v1.1.4), *tidyr* (v1.3.1), and *stringr* (v1.5.1).

Variants were classified as single nucleotide polymorphisms (SNPs) or insertions/deletions (INDELs) on the basis of allele string length. Specifically, a variant was designated as an SNP when both the reference and alternate alleles consisted of a single nucleotide (nchar (reference allele) = = 1 and nchar (alternative allele) = = 1), and as an INDEL otherwise. INDELs were further subclassified as insertions or deletions by comparing the character lengths of the reference and alternate allele strings, with insertions defined as nchar (Alt) > nchar (reference allele) and deletions as nchar (reference allele) > nchar (alternative allele).

Functional effect annotations, including missense variants, synonymous variants, upstream gene variants, and other predicted consequences, were extracted from the primary annotation field by splitting concatenated SnpEff entries on semicolons. Only the first annotation entry per variant was retained for downstream classification, corresponding to the highest-priority predicted effect assigned by SnpEff. High-confidence variants were identified by applying an alternative read depth threshold as a proxy for variant confidence, in addition to the upstream VCF-level quality filtering described above. Genes were subsequently ranked by their raw missense variant count to identify loci with the highest predicted functional mutational burden. The reference genome annotation for *Escherichia coli* strain NZ_CP014225.1 was retrieved from the NCBI database, and locus tags were joined to their corresponding gene names and protein product descriptions using the *locus_tag*, *gene*, and *product* fields, enabling functional interpretation of variant-affected loci. Final result tables were exported to Excel-formatted output files using the *writexl* package (v1.5.0).

*mcr-1* copy number analysis—*mcr-1* is plasmid-encoded and was absent from the chromosomal reference (NZ_CP014225.1) used in the original alignment, a dedicated plasmid mapping analysis was performed. Paired-end reads from strain AAS-14 were mapped to the canonical *mcr-1*-carrying plasmid KP347127.1 (64,015 bp) using Bowtie2 (v2. x, paired-end mode, 8 threads), followed by coordinate sorting and indexing with SAMtools. Mean read depth across the full plasmid was calculated using samtools depth and averaged with awk. The resulting plasmid mean depth was 331.5×, compared to a chromosome-wide mean depth of 321.3× (calculated from 200 randomly sampled genomic windows using pysam). This yields a plasmid-to-chromosome depth ratio of 1.03, which is indistinguishable from 1.0, representing no *mcr-1* gene duplication or elevated plasmid copy number. For comparison, all seven chromosomal 16S rRNA loci, which serve as internal single-copy calibration controls, showed depth ratios ranging from 0.85 to 1.62 (mean 1.08), consistent with the expected single-copy depth for chromosomal loci. The *mcr-1* ratio of 1.03 is within this range for a single copy gene.

#### Accession to the sequence data

The sequence data were deposited on April 27^th^ in the NCBI Sequence Read Archive (SRA) under BioSample accession SAMN57484683, as part of the BioProject PRJNA1457984.

### Statistical analysis

Each experiment was repeated 3–4 times. Statistical analysis was done using Microsoft Excel version 2501 and GraphPad Prism 9.3.1. One-way ANOVA and unpaired t-test were used for statistical analysis. Error bars represent standard error of mean between repeated experiments p-values are represented as follow (*P < 0.05, **P < 0.01, ***P < 0.001).

## Results

### Selected *E. coli* strains show striking resistance to colistin in comparison to the wild-type

**FIGURE 1:**
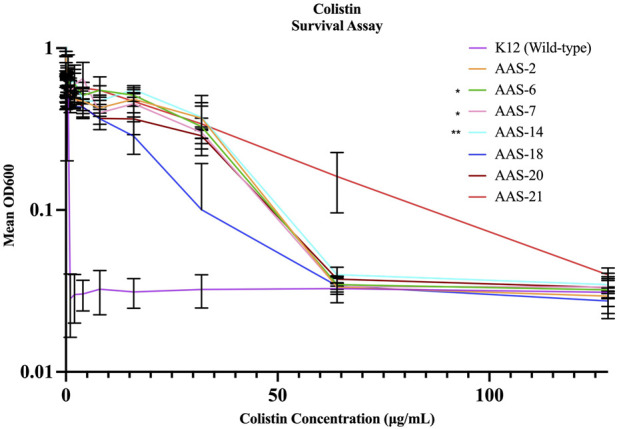
Survival analysis of the *E. coli* strains resistant to colistin in comparison to the wild-type *E. coli* (K12). Exponentially growing cultures were adjusted to an OD_600_ of 0.05 and allowed to grow at 37 °C overnight in MH media without and with increasing concentrations of colistin. The final OD_600_ was determined after 18–24 h of incubation. The figure is a representation of three independent determinations.

We examined whether colistin-resistant *E. coli* strains isolated from environmental samples would show differential responses to increasing concentrations of the antibiotic. Briefly, exponentially growing cells were diluted to an OD_600_ of 0.05 and treated with increasing concentrations of colistin, allowed to grow for 18–24 h, followed by the final measurement of the OD_600_. The strains tested all reached an OD_600_ between 0.8 and 1.0 in the absence of colistin (Figure 1). In the presence of colistin, the wild-type strain did not grow at 2.0 μg/mL, while the growth of strains AAS18 and AAS20 started to decline after 5.0 μg/mL and the other 5 strains AAS2, AAS6, AAS7, AAS14 and AAS21 began to diminish after 15.0 μg/mL and by 64.0 μg/mL these strains stop growing, except for strain AAS21 that continued to grow showing resistance beyond 100.0 μg/mL of the drug. These strains displayed IC_50_ values ranging from 12- to 55-fold higher, as compared to wild-type (Supplementary Table S1). Thus, it appears that strains AAS-6, -14, and −20 are the most resistant to colistin, followed by strains AAS-7 and -21, and strain AAS-18 with intermediate resistance.

### Colistin-resistant strains harbour the *mcr-1* gene

**FIGURE 2:**
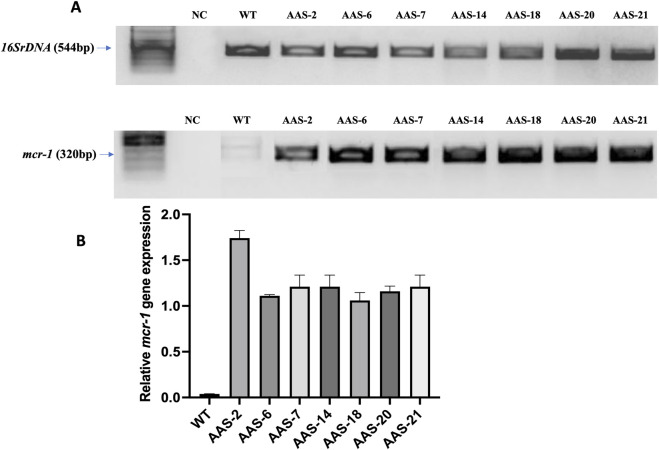
Detection of the *mcr-1* gene in the colistin-resistant strains. **(A)** Heat-denatured *E. coli* strains were directly used to amplify the control housekeeping gene, *16SrDNA*, and the *mcr-1* gene. NC, negative control contained water and the master-mix to rule out primer contamination. **(B)** Densitometric quantification of *mcr-1* gene expression relative to the control gene *16SrDNA*. The result is representative of three independent experiments. Error bars represent standard error of mean.

It is well established that the *mcr-1* gene is a key contributing factor causing resistance to colistin (Mondal et al., 2024; Uddin et al., 2022). As such, we checked whether the *mcr-1* gene would be carried by the colistin-resistant strains. In this experiment, template genomic DNA was prepared from single colonies of the indicated colistin-resistant and the wild-type strains and subjected to PCR amplification for the control housekeeping gene 16SrDNA (544 bp) and the colistin-resistant *mcr-1* gene (320 bp). All the strains showed amplification of the *16SrDNA* gene, and only the colistin-resistant strains showed amplification of the *mcr-1* gene (Figure 2). Since all the tested strains were positive for the *mcr-1* gene, it would appear that this gene might be the contributing factor for the colistin-resistance phenotypes.

### The colistin-resistant *E. coli* strains show cross-resistance to other antibiotic drug classes

**FIGURE 3:**
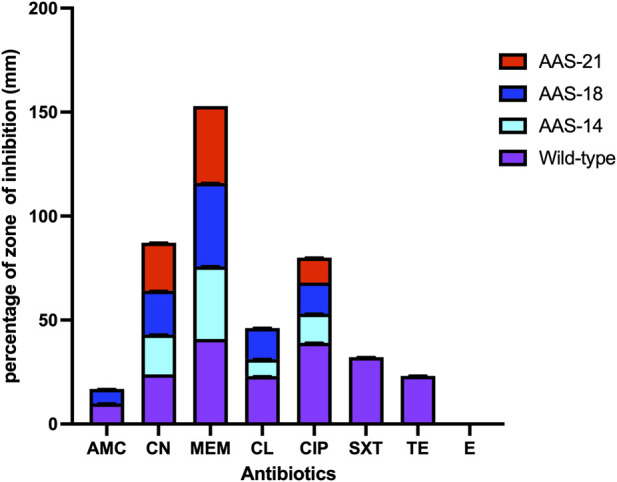
Antibiotic susceptibility testing of wild-type and selected *E. coli* colistin-resistant strains. Briefly, overnight culture of the strains grown in LB, were diluted to OD600 of 0.05 and spread onto MH solid media followed by the placement of the antibiotic containing disc. The bars represent the diameter (mm) obtained from the antibiotic susceptibility analyses of the indicated strains and reported as mean ± SD, zones of inhibition evaluated according to CLSI guidelines and interpretations. The figure is a representation of three independent determinations. Amoxicillin (AMC), Gentamicin (CN), Meropenem (MEM), Cephalexin (CL), Ciprofloxacin (CIP), Sulfamethoxazole (SXT), Tetracycline (TE), and Erythromycin (E).

We examined if the colistin-resistant strains would display resistance to antibiotics from various classes that include **amoxicillin** from the beta-lactams family, **gentamicin** from the aminoglycosides family that inhibits the 30S ribosome, **meropenem** from the carbapenems family used for treating serious infections, **cephalexin** from the cephalosporins family, **ciprofloxacin** from the fluoroquinolones family that inhibits DNA gyrase activity, **sulfamethoxazole** from the sulfonamides family that block folate synthesis, **tetracycline** from the tetracyclines family that acts as broad spectrum protein synthesis inhibitor and inhibits the 30S ribosome, and **erythromycin** from the macrolides family that inhibits the 50S ribosome. The latter is generally not used as nearly all tested *E. coli* isolates are known to be intrinsically resistant to erythromycin, and therefore showed no zone of inhibition due to outer membrane barrier and efflux pumps that can prevent intracellular accumulation of the antibiotic (Melander et al., 2023). As such, we used erythromycin as a negative control. To conduct the antimicrobial susceptibility test, we used the standard method of disc diffusion. The wild-type *E. coli* showed striking sensitivity to gentamicin, meropenem, cephalexin, ciprofloxacin, sulfamethoxazole, and tetracycline, and to a lesser extent to amoxicillin, but displayed no sensitivity to erythromycin (no zone) (Figure 3, and see Supplementary Figure S1, supplementary data on the disc assays). In contrast, the colistin-resistant strains tested, AAS-14, AAS-18, and AAS-21, exhibited resistance to sulfamethoxazole and tetracycline while retaining wild-type sensitivity to gentamicin and meropenem. Strains AAS-14, AAS-18 and AAS-21 also showed reduced sensitivity or no sensitivity to cephalexin, ciprofloxacin, and amoxicillin as compared to the wild-type strain (Figure 3). It would appear that the colistin-resistant strains acquired additional genes to counter the effects of cephalexin, ciprofloxacin, sulfamethoxazole, and tetracycline, although in the case of strain AAS-21, the acquired gene conferred full resistance to cephalexin (Figure 3).

In separate experiments, the exponentially growing colistin-resistant strains AAS-14, AAS-18, and AAS-21, and the wild-type were diluted to an OD_600_ of 0.05 and treated with increasing concentrations of tazocin, a member of the beta-lactam family, and meropenem, and allowed to grow for 18–24 h followed by the measurement of the final OD_600_. The colistin-resistant strains were very sensitive to tazocin and, similar to the disc assays, were also very sensitive to meropenem, as the wild-type (see Supplementary Figure S2). The sensitivities of the strains to tazocin and meropenem were not statistically different from the wild-type (Supplementary Figure S2). Thus, even though strains AAS-14, AAS-18, and AAS-21 are very resistant to colistin, their growth can be completely suppressed by alternative classes of antibiotics.

### The colistin-resistant strains exhibit differential resistance to ultraviolet light, UVC

**FIGURE 4:**
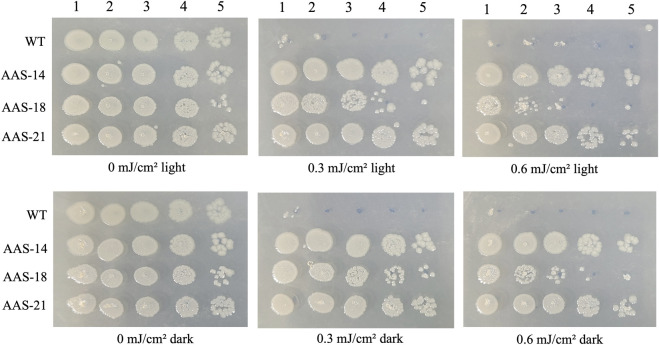
Spot test analysis showing the resistance of the *E. coli* strains to UVC. Exponentially growing cells were adjusted to an OD_600_ of 0.1, serially diluted to 10,000-fold, and 4 µL spotted onto the solid LB plates. The numbers above each strain represent the following dilutions: 1, undiluted (OD600 of 0.1); 2, 10-fold; 3, 100-fold; 4, 1000-fold; and 5, 10,000-fold. Light, plates were placed in an incubator with a glass window to allow ambient light transmission. Dark, following UVC exposure, the plates were covered with aluminum foil and placed in an incubator with no light transmission. Plates were photographed after 18–24 h of incubation at 37 °C using a mobile phone. The result is representative of three independent experiments.

Bacterial strains living in harsh environmental conditions are constantly exposed to ultraviolet light emanating from sunlight, which can damage the DNA, causing mutations and allowing the cells to become UV-resistant. In addition, UVC is used for destroying pathogens during wastewater management (Kato and Kansha, 2024). As such, we checked whether the strains would show resistance to ultraviolet light. Briefly, exponentially growing cultures were adjusted to an OD_600_ of 0.1, serially diluted to 10,000-fold, and a fixed volume of 4 µL of each dilution was spotted onto solid LB agar plates, followed by exposure to UVC and incubation of the plates in either ambient light or complete darkness. The wild-type cells were extremely sensitive when exposed to the UVC dose of 0.3 mJ/cm^2^, while strains AAS-14 and AAS-21 were unaffected, and AAS-18 displayed sensitivity that was more prominent at the higher UVC dose of 0.6 mJ/cm^2^ (Figure 4). Surprisingly, the three colistin-resistant strains appeared to be slightly more resistant to the UVC doses when incubated in the dark as opposed to the ambient light, suggesting that the strains possess an efficient mechanism to process UVC-induced DNA lesions that could be more active in the dark.

### The colistin-resistant strains are hyper-resistant to the chemical oxidant H_2_O_2_ and express wild-type levels of catalase activity

**FIGURE 5:**
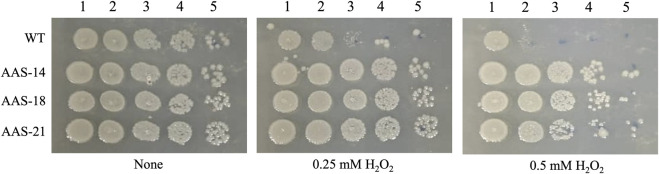
Spot test analysis showing H_2_O_2_-resistance of the *E. coli* strains. Exponentially growing cultures were adjusted to an OD_600_ of 0.1, serially diluted to 10,000-fold, and 4 µL of each dilution spotted onto LB agar plates with either 0, 0.25, or 0.5 mM H_2_O_2_. The numbers above each strain represent the following dilutions: 1, undiluted (OD600 of 0.1); 2, 10-fold; 3, 100-fold; 4, 1000-fold; and 5, 10,000-fold. The plates were incubated at 37 °C for 18–24 h, before being photographed using a mobile phone. The result is representative of three independent experiments.

Several DNA-damaging agents are used for wastewater treatment, and these include the powerful oxidant hydrogen peroxide (H_2_O_2_), hypochlorite, potassium permanganate, and ultraviolet light (Kato and Kansha, 2024). Since the *E. coli* strains were isolated from treated wastewater, we checked whether the colistin-resistant bacteria would display resistance to H_2_O_2_. Exponentially growing cultures were adjusted to an OD_600_ of 0.1, serially diluted to 10,000-fold, and a fixed volume of 4 µL of each dilution was spotted onto plates containing either 0, 0.25, or 0.5 mM H_2_O_2_. All four strains, wild-type, AAS-14, AAS-18, and AAS-21, grew on the plate without H_2_O_2_. In contrast, only strains AAS-14, AAS-18, and AAS-21 retained nearly normal growth on plates with 0.5 mM H_2_O_2_, while the wild-type barely grew (Figure 5). At higher H_2_O_2_ concentration, above 0.5 mM, strain AAS-14 appeared to be slightly more resistant to the oxidant than strains AAS-18 and AAS-21, while the wild-type did not grow.

We performed an independent analysis using gradient plate assays, whereby exponentially growing cultures were imprinted along a gradient of either H_2_O_2_ or another oxidant, menadione. Menadione undergoes redox cycling and generates reactive oxygen species, primarily superoxide radicals, causing DNA damage and leading to cell death (Ramotar et al., 1991). The gradient plate analysis revealed that strains AAS-14, AAS-18, and AAS-21 were indeed hyper-resistant to H_2_O_2_ as compared to the wild-type strain, and that strain AAS-14 was more resistant to H_2_O_2_ than the other two strains AAS-18 and AAS-21 (Supplementary Figure S3). In addition, strains AAS-14 and AAS-18 showed resistance to menadione while strain AAS-21 showed nearly the same level of resistance to the drug as compared to the wild-type (Supplementary Figure S3). In the control experiment, all four strains grew along the entire gradient plate that contained no oxidant (Supplementary Figure S3). Collectively, the data indicated that the colistin-resistant strains may have acquired additional genes(s) to enhance resistance to the oxidants or the genomes of the strains have been mutated to combat the genotoxic effects of the oxidants.

**FIGURE 6:**
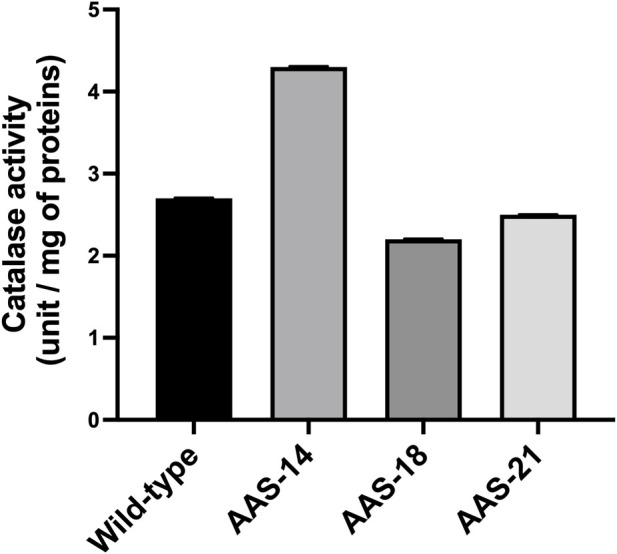
Comparison of the level of catalase activity in the wild-type and the H_2_O_2_-resistant strains. Briefly, total extract was prepared from exponentially growing cultures and the same amount of proteins from each extract was used to determine the catalase activity by monitoring the decrease in A240 absorbance of H_2_O_2._ The values were the averages from three independent experiments.

We next check if the increased resistance of the strains towards H_2_O_2_ would be correlated with the enzymatic activity of catalase, which is known to decompose H_2_O_2_. Total extracts were prepared from exponentially growing cultures of the wild-type and the three H_2_O_2_-resistant strains and quantified for catalase activity by monitoring the decrease in H_2_O_2_ initial absorbance at OD_240_ nm over a duration of 5 min. The analysis revealed that the H_2_O_2_-resistant strain AAS-14 possessed at least 1.6-fold higher level of catalase activity, while the other two resistant strains AAS-18 and AAS-21 harboured nearly the same level as the wild-type strain (Figure 6). Since all three strains AAS-14, -18, and −21 conferred the same extent of resistance to H_2_O_2_ (Figure 5), it is unlikely that the enhanced catalase activity in strain AAS-14 contributed to the increased resistance to H_2_O_2_.

### Strain AAS-14 is proficient in protecting its genome from H_2_O_2_-induced DNA lesions as compared to the wild-type

We have previously shown that *E. coli* strains challenged with H_2_O_2_ can accumulate H_2_O_2_-induced chromosomal DNA lesions, such as strand breaks that can support DNA repair synthesis using an assay that employed the incorporation of [methyl- ^3^H]-dTTP by DNA polymerase1 into the chromosomal (Ramotar et al., 1991). For this experiment, we adopted a different approach to avoid using radioactive material by analyzing on a DNA agarose gel chromosomal DNA extracted from untreated, H_2_O_2_-treated, and H_2_O_2_-treated and recovered cells. The analysis revealed that chromosomal DNA extracted from exponentially growing H_2_O_2_-treated WT cells (50 mM for 30 min) was fragmented, and there appeared to be minimal restoration of the chromosomal DNA following the treatment and recovery in fresh media for 2 h. In contrast, there was no visible fragmentation with the chromosomal DNA isolated from the exponentially growing strain AAS-14 treated with H_2_O_2_. We suggest that strain AAS-14 may harbour mutations that allow it to efficiently repair H_2_O_2_-induced DNA lesions, thus conferring resistance to the oxidant.

### Whole genome sequence reveals unique differences between the colistin-resistant strain and wild-type

**FIGURE 7:**
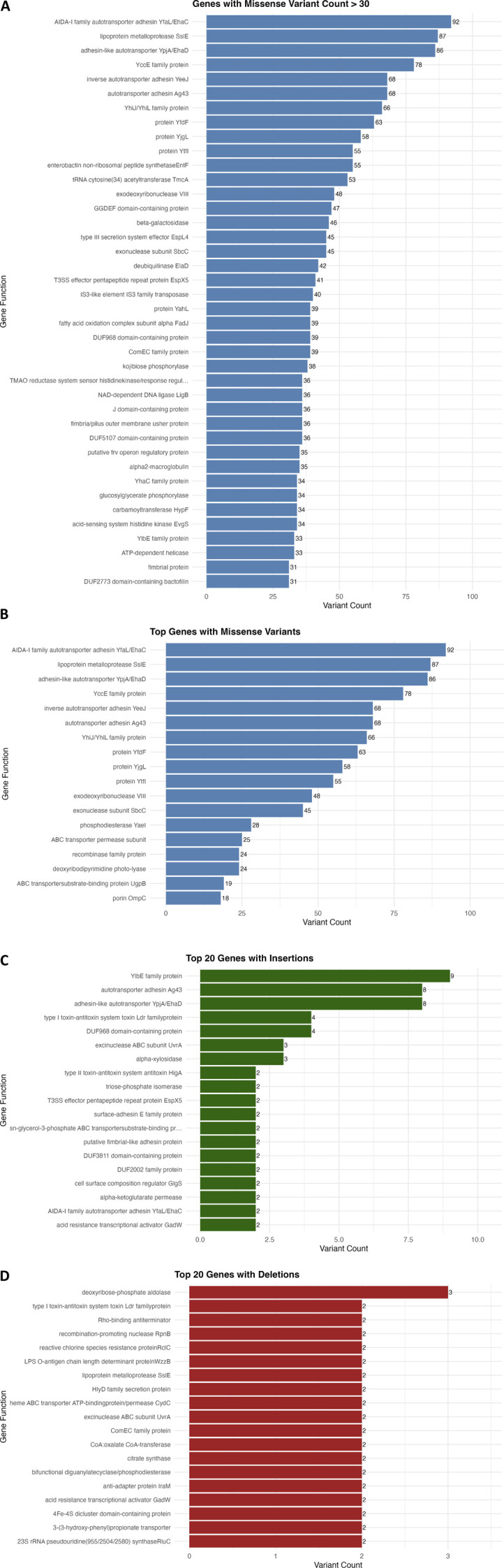
Comparison of the genome sequence of strain AAS-14 with the wild-type *E. coli* K12 reveals several genes with multiple missense, insertion, and deletion mutations. The bars represent the number of mutations in a particular gene and are sorted from highest to lowest. **(A)** Top genes with missense mutations greater than 30 (see supplementary PDF for upto 100 genes); **(B)** top genes with missense variants **(C)**, top 20 genes with insertion mutations; and **(D)**, top 20 genes with deletion mutations.

We next performed whole genome sequence analysis of three colistin-resistant strains AAS-14, AAS-18, and AAS-21, to gain insights into possible genes that could account for their phenotypes. The analysis identified several genes with multiple missense variants, and genes with two to three insertion and deletion mutations (Figures 7A‐D; see also Supplementary Material with high resolution Figures as pdf and Excel files with functional roles of the genes that have been mutated). Some of these genes encode adhesion proteins, as well as DNA repair proteins involved in the repair of UVC-induced DNA lesions and DNA single- and double-strand breaks. In addition, the DNA sequence analysis also revealed that, for example, strain AAS-14 harboured several common and distinct genes among the strains (Supplementary Table S2). The strains showed distinct multi-locus sequence typing (ST10, ST770, ST3489) and serotypes. ST10, for example, in strain AAS‐18 has been associated with pathogenic *E. coli* and reported to be a common cause of urinary tract infections in Qatar (Eltai et al., 2018). ST770 in strain AAS-14 is associated with environmental isolates, and ST3489 in strain AAS-21 is rarely detected but associated with animals, suggesting an emerging lineage and incidental colonization.

Using the tool Virulence Finder, several virulence genes carried by strain AAS-14 had high identity (98%–100%) and coverage, which directly correlates with high pathogenicity (91.7%). This percentage is driven by the synergistic existence of virulence factors that facilitate immune evasion and damage in host tissue. A key contributing factor increased serum survival gene (*iss*), outer membrane protein (*traT*), which causes complement lysis resistance and persistence within the host. In addition, pore-forming toxins such as *hlyF* and *hlyE* cause tissue lysis and degradation, iron-acquisition system factors, *iutA*, *iucC*, and *ChuA*, which allow bacterial growth in iron-restricting environments like the human blood. Other colonization factors also exist, like *papA*, *papC*, *fimH*, which form fimbrial structures and adhesion, all these factors allow persistence and growth within the host and therefore differentiate the highly pathogenic strain.

The genotypic and phenotypic resistance of AAS-14 showed consistency; from detected ARGs, *qnrB4* and *mcr-1* account for resistance to ciprofloxacin (Figure 3) and colistin, respectively. In addition, the presence of *Sul1*, *Sul2*, *dfrA1*, and *dfrA7* accounts for the resistance to the agents, trimethoprim and sulfonamide. This co-occurrence explains the resistance exhibited to sulfamethoxazole (Figure 3), *tet(M)* and *tet(A)* genes account for the resistance to tetracycline (Figure 3). Gentamicin-specific modifying enzymes (16S-rRNA methylases, *aac(3)*, *aac(6′)-lb* were not detected, this explains the susceptibility shown by strain AA-14 to gentamicin (Figure 3). The ARGs detected do not consist of genes related to carbapenemase activity, which is consistent with susceptibility to meropenem. In addition, strain AAS-14 carried at least four distinct genes (*aadA3*, *blaDHA-1*, *blaTEM-1B*, and *qnrB4*) that are not carried by strains AAS-18 and AAS-21 (Supplementary Table S2). Strain AAS-21 carried a unique gene *floR* encoding an efflux pump that removes chloramphenicol from the cells, which was not present in strain AAS-14 and AAS-18 (Supplementary Table S2).

Moreover, the analysis revealed that the strains harbored diverse elements, including genomic islands, insertion sequences, integrons, chromosomal elements, transposons, and plasmids (Supplementary Tables S2–S6). For example, strain AAS-14 (Supplementary Table S4) carried the largest number of genetic elements, various clusters of elements constituting resistance and virulence, a diverse set of ARGs across plasmids and integrons, and with high virulence (type III secretion system genes, capsular synthesis genes, and adhesins (Supplementary Table S7), as well as the presence of plasmids carrying the virulence capability of the strains, in comparison to the wild-type strain (Supplementary Table S3). Some of the virulence genes encode proteins that maintain adhesion, colonization, horizontal gene transfer, iron acquisition, transport, and metabolism (Supplementary Table S7).

## Discussion

In the present study, we obtained *E. coli* strains that were deemed to be resistant to the antibiotic colistin but were not further characterized. Colistin is considered a last-resort antibiotic for treating multidrug-resistant gram-negative bacteria such as carbapenem-resistant *Klebsiella pneumoniae* (Falagas et al., 2008). Despite the effectiveness of colistin and its classification by the World Health Organization as an essential drug for preventing the spread of bacterial infections, it has several side effects that include causing kidney toxicity and neurological problems, and even more severe side effects such as anaphylaxis. A major clinical problem is that some infectious bacteria show a diverse range of resistance to colistin, and this has become a challenge to treat infected patients with the drug without causing, for example, kidney toxicity. Herein, we obtained several colistin-resistant *E. coli* strains isolated from a mixture of wastewater (Khine et al., 2023), characterized seven of the strains, and uncovered that they exhibited different half-maximal inhibitory concentration (IC_50_) values ranging from 12- to 55-fold higher than the Wild-type. We envision that strains exhibiting a 55-fold increase in colistin resistance compared to the wild-type warrant further characterization, as such the resistance levels would likely exceed the clinically achievable plasma concentrations of colistin, which are typically in the range of ∼ 2–4 μg/mL in treated patients (Sanabria et al., 2021). Of note, since some of the colistin-resistant strains were found in the effluent wastewater used for crop irrigation, it is likely that these drug- and chemical-resistant strains can contaminate leafy greens such as lettuce, spinach, and broccoli that are often consumed raw. As such, these highly drug-resistant strains that enter the food chain can colonize the intestines of humans leading to infectious diseases. Thus, patients infected with these highly colistin-resistant environmental strains are at greater risk and unable to achieve clinical resolution with the maximal dose that can be given (Sanabria et al., 2021). Consequently, the spread of colistin resistance poses a serious public health threat by undermining one of the last-resort antibiotics and highlighting the need for integrated One Health surveillance and antimicrobial stewardship strategies.

Of the seven strains, AAS-14 had one of the highest IC_50_ values, while AAS-18 showed an intermediate value as compared to the wild-type (Supplementary Table S1). We further showed that the colistin resistance can be explained by the presence of the plasmid-borne (mobile colistin resistance) *mcr-1* gene that spreads horizontally, and encodes the small enzyme phosphoethanolamine transferase that adds a phosphoethanolamine group to lipid A thereby decreasing colistin binding to the bacteria. The seven strains tested all harbored the same *mcr-1* gene, and not any of the other nine members (*mcr-2* to *mcr-10*), and we found no evidence that the *mcr-1* gene is overexpressed, for example, in strain AAS-14 to account for the higher IC_50_ values. Moreover, since all the strains sheltered the same *mcr-1* gene, the increased resistance of some strains cannot be explained by variant forms of the gene. At the moment, we do not know what other factors, besides *mcr-1*, the strains are carrying that could account for the increased resistance to colistin. There are other known genes that are embedded in the chromosome that could contribute to colistin resistance, and these include the *basR/basS* operon, which, when mutated, can increase lipid A modification by the addition of phosphoethanolamine (Janssen and van Schaik, 2021). Additionally, mutations in the endogenous genes such as *gyrA* (topoisomerase involved in DNA replication), *phoQ*, and *acrA* (drug efflux pump) have been previously reported to contribute partially to colistin resistance (Bakleh et al., 2024). Indeed, we examined our WGS data specifically for variants in the PhoPQ and PmrAB regulatory loci, which are two-component regulatory systems in Gram-negative bacteria that act as sensory mechanisms, detecting environmental threats and adapting for survival. For example, these two-component systems primarily regulate LPS modification, protecting the bacteria from cationic antimicrobial compounds such as colistin. We identified five non-synonymous missense mutations in *pmrB* (Val354Ile, Asp286Gly, Thr249Ile, Ser141Asn, His5Arg; all QUAL>30, PASS-filtered) and one missense mutation in *phoP* (Ile44Leu). These chromosomal mutations could directly regulate lipid A modification and colistin susceptibility, likely act synergistically with the plasmid-encoded *mcr-1* gene to account for the exceptionally high MIC observed by strain AAS-21. This finding is consistent with the literature showing that PmrB gain-of-function variants combined with *mcr-1* can elevate colistin MICs well beyond the range conferred by *mcr-1* alone (Huang et al., 2021; Zhu et al., 2021; Luo et al., 2019). It is noteworthy that although strains AAS-14 and AAS-21 have similar resistance to colistin (Figure 1), the strains are distinct. For example, strain AAS-14 showed sensitivity to cephalexin, while strain AAS-21 was completely resistant to the antibiotic (Figure 3). From our data above and evidence from the literature, we believe that there must be other genes, whether it is plasmid-borne genes or mutated chromosomal loci, that would allow *E. coli* to become extremely resistant to colistin. These genes could also involve defective uptake transporters and/or activated efflux pumps.

**FIGURE 8:**
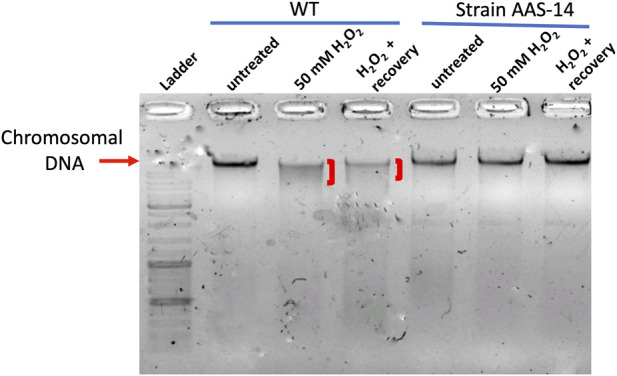
H_2_O_2_-induced DNA fragmentation in the wild-type strain and not in strain AAS-14. Briefly, exponentially growing cells were either untreated, treated 2 h in fresh media without H_2_O_2_, or treated with H_2_O_2_ and recovered for 2 h in fresh media with H_2_O_2_. The chromosomal DNA was extracted from each condition and analyzed by 1.0% DNA agarose gel electrophoresis. The red arrow indicates the position of the extracted chromosomal DNA, and the red brackets show the fragmentation of the chromosomal DNA. The figure is representative of three independent experiments.

We were interested in determining whether the strains also developed resistance to the chemicals used for wastewater management to destroy pathogens before the treated water is discharged or recycled. Some of these chemical agents include sodium hypochlorite (household bleach), potassium permanganate, and H_2_O_2_, which all have in common the ability to produce reactive oxygen species that can directly damage DNA to produce a broad range of mutagenic DNA lesions that include 8-oxo-guanine, DNA single and double-strand breaks (Masson and Ramotar, 1997). In addition, these chemicals, such as sodium hypochlorite, react with organic compounds in wastewater to produce chloroamines that can alkylate DNA bases, leading to mutations, and H_2_O_2_ can react with heavy metal salts such as ferric chloride via the Fenton reaction to generate potent hydroxyl radicals that can directly damage DNA (Mendez-Garrido et al., 2014; Imlay and Linn, 1987; Imlay and Linn, 1988). Thus, bacteria can mutate during wastewater treatment and survive to become resistant to the chemicals. As such, we examined whether the colistin-resistant strains acquired resistance to two different oxidants, H_2_O_2_ and menadione. We were surprised to find that some of the strains were extremely resistant to H_2_O_2_, that is, exceeding 0.5 mM, when the wild-type strain was killed. Of the three strains tested, AAS-14 was the most resistant to H_2_O_2_. In addition, the H_2_O_2_ treatment caused no detectable DNA damage to strain AAS-14 as compared to the wild-type strain (Ramotar et al., 1991). One possible interpretation of our findings is that strain AAS-14, and extrapolated to strains AAS-18 and AAS-21, acquired an elevated capacity to repair H_2_O_2_-induced DNA lesions, causing the strains to be hyper-resistant to the oxidant. Several enzymes exist in *E. coli* that can directly repair H_2_O_2_-induced DNA single-strand breaks to allow DNA repair synthesis (Masson and Ramotar, 1997). These enzymes include the base-excision DNA repair proteins exonuclease III (encoded by *xth*) and endonuclease IV (encoded by *nfo*) that possess the ability to remove H_2_O_2_-induced DNA single strand breaks containing blocked 3′-ends (Ramotar et al., 1991). *E. coli* mutants lacking both of these genes, *xth* and *nfo*, are very sensitive to H_2_O_2_, although it is not known whether overexpression of *xth* and *nfo* together would confer enhanced resistance to H_2_O_2_ (Ramotar et al., 1991). In addition to *xth* and *nfo*, *E. coli* possesses the gene *recE* encoding the 5′ to 3′ exodeoxyribonuclease VIII, which can channel H_2_O_2_-induced DNA lesions into the recombinational DNA repair pathway (Figure 8) (Galhardo et al., 2000). Likewise, *E. coli* harbors in its genome the genes *sbcC* and *sbcD* encoding a two-subunit complex consisting of an ATPase activity. A 5′ to 3′ exonuclease, and a 3′ to 5′ endonuclease that can act on single-stranded DNA (Figure 8) (Goswami and Gowrishankar, 2022; Wendel et al., 2018). This latter enzyme complex can rapidly repair DNA single-strand breaks using the homologous recombinational DNA repair pathway (Goswami and Gowrishankar, 2022). At the moment, we are checking if any of the genes encoding these and other potential DNA repair enzymes are mutated such that they produce variant proteins that acquire gain-of-function allowing the colistin-resistant bacteria to become highly resistant to H_2_O_2_. We cannot exclude alternative mechanisms that could account for the hyper-resistance of strain AAS-14 to H_2_O_2_, and one such mechanism could be due to limited iron uptake or its intracellular availability, and therefore, it cannot drive the Fenton reaction to produce the genotoxic hydroxyl radical species to damage the DNA. Other mechanisms that could play a role in the hyper-resistance of strain AAS-14 to menadione and H_2_O_2_ may involve the SoxRS and the OxyR transcription factors that serve as molecular detectors to defend against oxidative stress (Mendez et al., 2022). SoxRS is activated by superoxide while OxyR is activated by H_2_O_2_. We screened our WGS data for mutations in the *soxRS* and *oxyR* genes and found no missense mutations or mutations in the promoter regions of these genes that would upregulate their function and confer enhanced resistance to menadione or H_2_O_2_. As such, we exclude the functions of SoxRS and OxyR, respectively, in the hyper-resistance of strain AAS-14 to menadione and H_2_O_2_.

At the moment, we do not have an explanation for the UVC-resistance displayed by strains AAS-14, -18 and −21. Some of the key genes encoding proteins involved in repairing UVC-induced DNA lesions such as DNA photolyase and UvrA, respectively, contained 24 missense mutations (*phrA*) and three insertions and two deletions (*uvrA*) in strain AAS-14 (see Supplementary Material I). Since neither the *phrA* nor *uvrA* gene is known to possess mutations that lead to gain-of-function and that deletion of these genes are known to sensitize *E. coli* to UVC, it is counterintuitive that the mutated *phrA* and *uvrA* genes contribution to the UVC-resistance. A plausible explanation is that DNA damage recognition by the variant UvrA may remain tightly bound to pyrimidine dimers created by UVC, preventing nucleotide excision repair, and this signals activation of the SOS response, upregulating multiple gene products including RecA to promote recombinational repair of the UVC-induced DNA lesions (Ghodke et al., 2020; Sass and Lovett, 2024; Ghosh et al., 2025).

In summary, we have shown that *E. coli* isolated from wastewater have complex resistance patterns to drugs, showing resistance to various antibiotics by acquiring plasmid-borne antibiotic resistance genes through horizontal transfer. However, it remains unclear why some of the colistin-resistant strains conferred higher levels of resistance to the drug. Of note, we did not anticipate finding that some of the bacteria would show cross-resistance to chemical oxidants, in particular, H_2_O_2_. Since H_2_O_2_ is a DNA-damaging agent and a mutagen that can generate mutagenic DNA lesions via the production of hydroxyl radicals in the presence of iron, we believe that H_2_O_2_ resistance of the strains may arise as a result of mutations that activate DNA repair mechanisms. We further believe that H_2_O_2_-treatment in wastewater management would allow bacteria to mutate and acquire resistance to any compounds present in the milieu, such as antibiotics from hospital wastewater, heavy metals from industrial wastewater, and secondary compounds formed from hypochlorite reactions with organic amines from municipal wastewater. It is not surprising that previously well-defined antibiotic resistance genes can gain multiple mutations to acquire cross-resistance to other antibiotics. Moreover, wastewater management to rid pathogenic bacteria with H_2_O_2_ will give rise to even more infectious strains, and as such, it is necessary to find alternative approaches that avoid the use of genotoxic agents.

## Data Availability

The datasets presented in this study can be found in online repositories. The names of the repository/repositories and accession number(s) can be found in the article/Supplementary Material.
