# Supplementary material for: Biochemical and genomic characterization of last-resort antibiotic resistance and oxidant tolerance in environmental E. coli
Source: Front Toxicol. 2026 Jun 3;8:1822843. doi: 10.3389/ftox.2026.1822843 (PMC13271735; doi:10.3389/ftox.2026.1822843)
Supplement: Supplementary file 1 [file Supplementaryfile1.docx]

**Supplementary Figures and Tables**


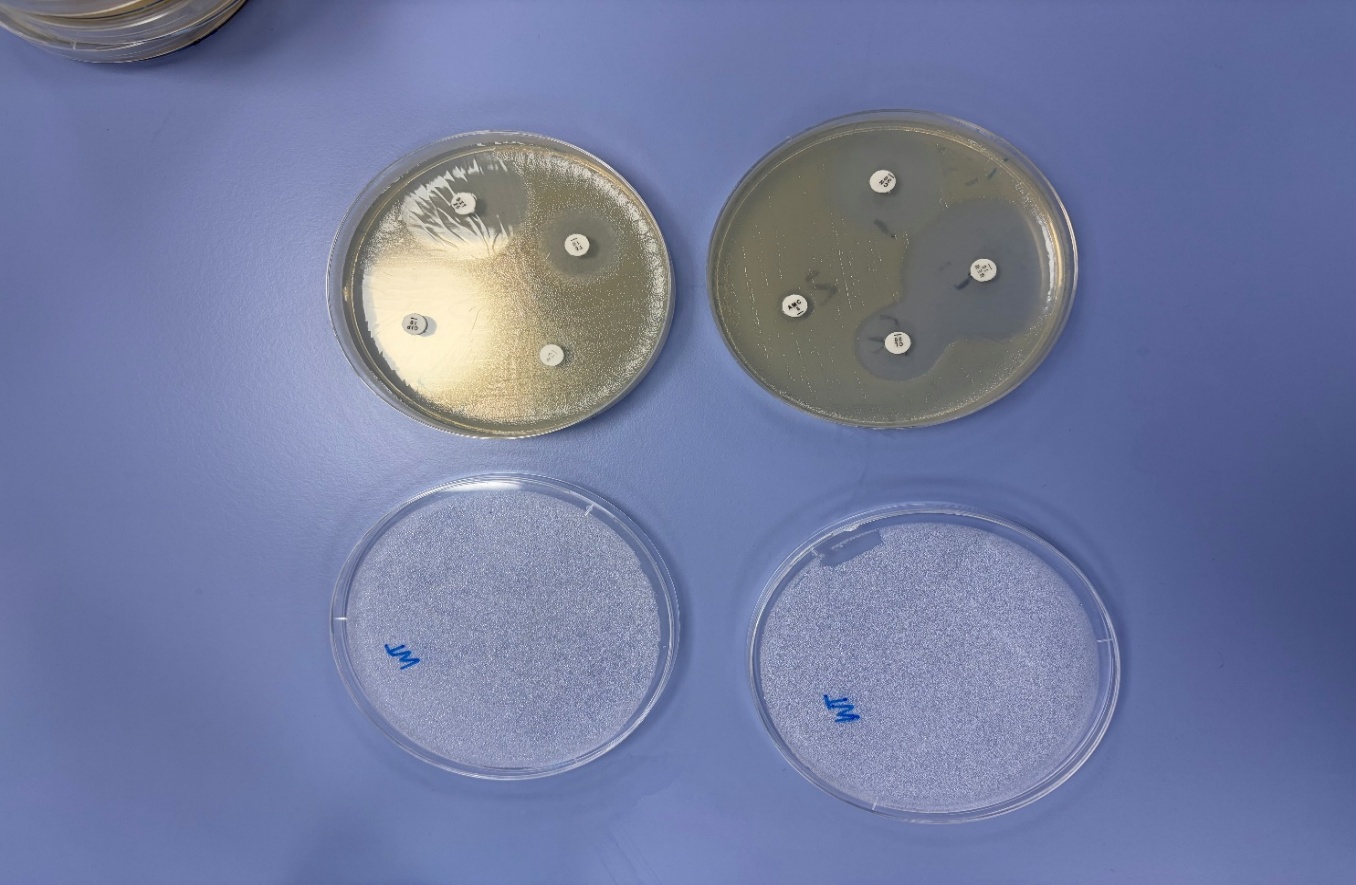

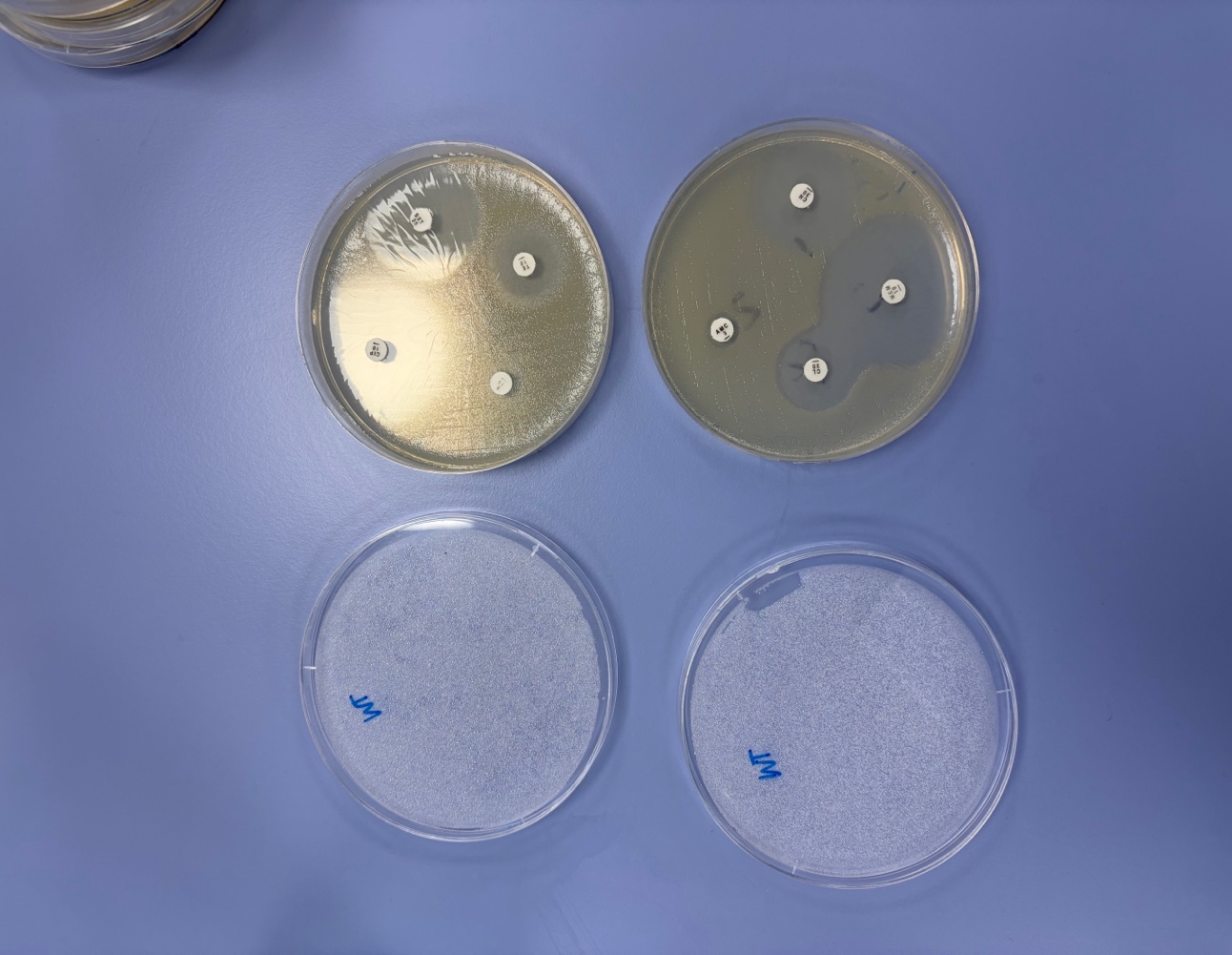

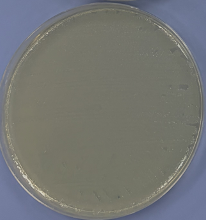

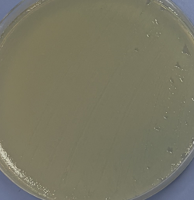

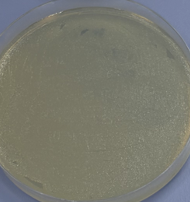

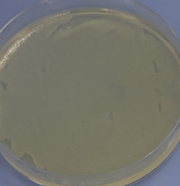

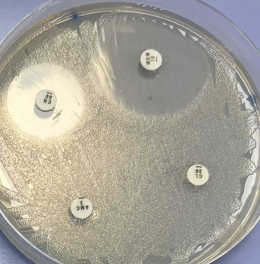

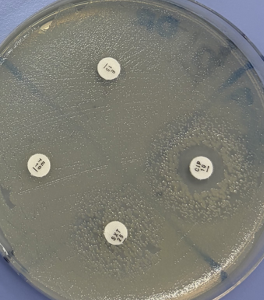

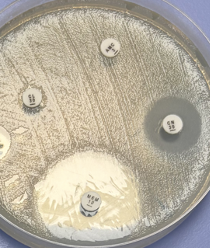

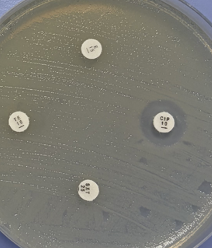

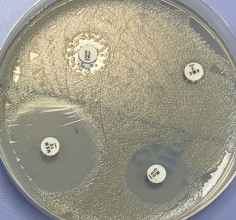

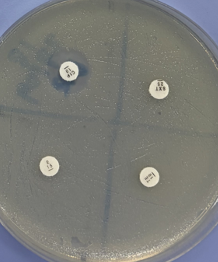


WT

AAS-14

AAS-18

AAS-21

Ciprofloxacin

Sulfamethoxazole

Sulfamethoxazole

Ciprofloxacin

Tetracyclinev

Tetracycline

Tetracyclinev

**Figure S1. Showing the antibiotic susceptibility testing of the wild-type and the antibiotic-resistant strains.** Exponentially growing cells at an OD_600_ of 0.05 were plated as a lawn onto MH agar, and the discs were placed directly onto the lawn of bacteria. Pictures of the plates were taken 18-24 hours post incubation. The cyan colored circle indicates sensitivity to meropenem. The distances in mm are plotted in Fig. 3.

**
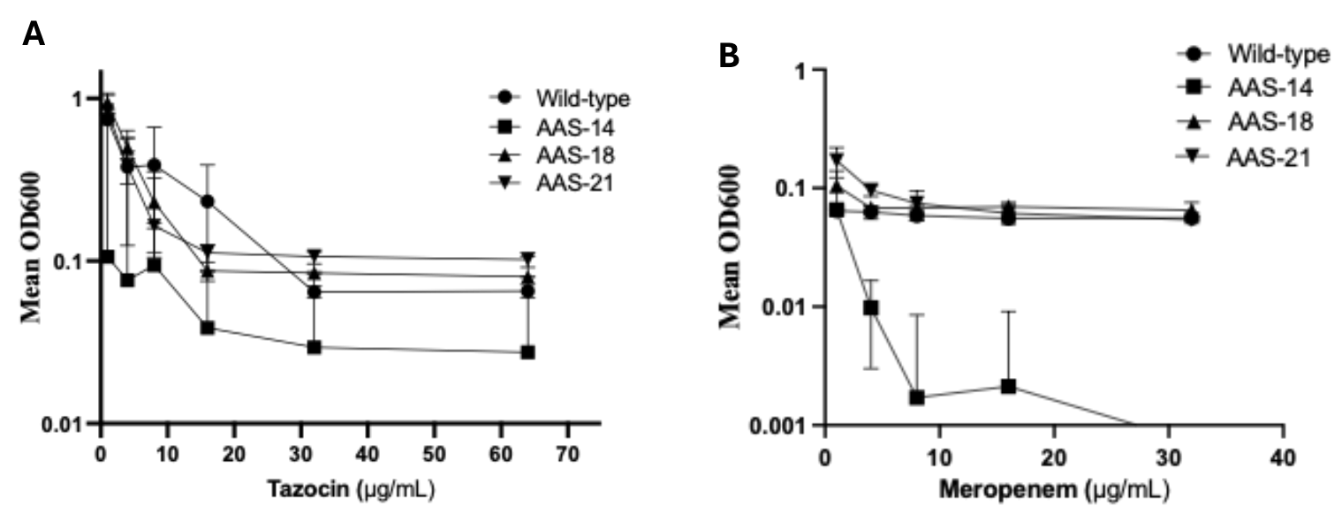
**

**Figure S2. Plots showing the sensitivity of the wild-type and the antibiotic-resistant strains AAS-14, -18, and 21 towards (A) Tazocin and (B) Meropenem.** The analysis was conducted as described in Figure 1.

**
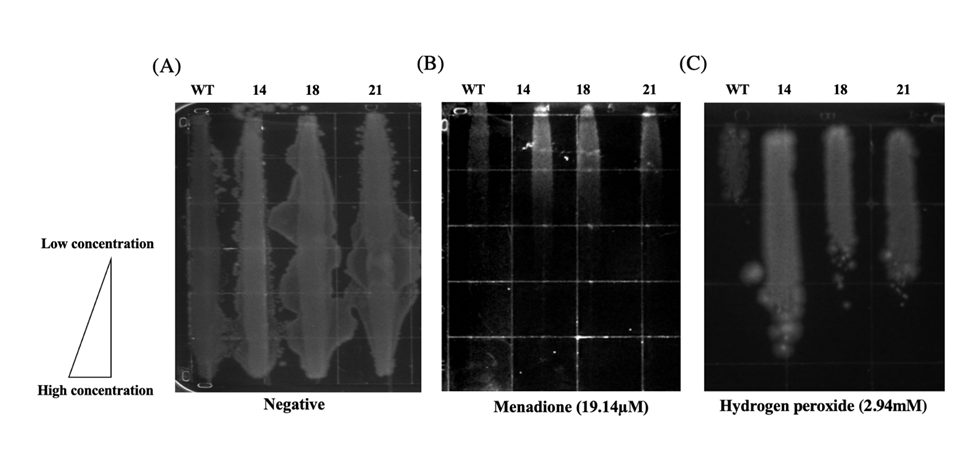
**

**Figure S3. Resistances conferred by the Wild-type and the indicated antibiotic-resistant strains.** Panels, initial amount of agent in the bottom layer of the gradient: (A) control, no drug, (B) 19.14 µM menadione, and (C) 2.94 mM hydrogen peroxide

**Table S1. Half maximal inhibitory concentration (IC_50_) of colistin for each tested *E. coli* strain**.

| ***E. coli* strain** | **Colistin IC_50_ (µg/mL)** ^a^ |
| --- | --- |
| Wild-type | 0.5 |
| AAS-2 | 6.47 |
| AAS-6 | 27.29 |
| AAS-7 | 20.91 |
| AAS-14 | 26.78 |
| AAS-18 | 7.63 |
| AAS-20 | 23.91 |
| AAS-21 | 12.94 |

^a^ Measurements based on mean OD of three replicates of strains grown in MH media, with IC_50_ range of 0.5 µg/mL for wild-type, and highest for strains AAS-6 (27.29 µg/mL) and AAS-14 (26.78 µg/mL).

**Table S2. Comparison of the MLST types, pathogenicity, serotypes, CH-Types, AMR genes, AMR phenotypes, and virulence genes using whole genome sequencing data.**

| **Isolate** |  | **MLST Type** | **Pathogenicity** | **Serotype** | **CH-Type** | **AMR Genes** | **AMR Phenotype** | **Virulence Genes** |
| --- | --- | --- | --- | --- | --- | --- | --- | --- |
| **K12 Wild-type** |  | ST10 | 87.5% | O16:H48 | fumC11, fimH27 |  |  | AslA, csgA, fimH, gad, hlyE, iss, nlpl, ompT, terC, yehA, yehB, yehC, yehD |
| **AAS-14** |  | ST770 | 91.7% | O25:H51 | fumC116, fimH552 | aadA1, aph(3')-Ia, aph(3'')-Ib, aadA3, blaDHA-1, blaTEM-1B, mcr-1.1, fosA4, mph(A), qnrB4, sul1, sul2, tet(M), tet(A), dfrA1, dfrA7 | Spectinomycin, streptomycin, neomycin, kanamycin, lividomycin, paromomycin, ribostamycin, an unknown aminoglycoside, amoxicillin, amoxicillin+clavulanic acid, ampicillin, ampicillin+clavulanic acid, cefotaxime, cefoxitin, ceftazidime, piperacillin, piperacillin+tazobactam, ticarcillin, ticarcillin+clavulanic acid, cephalothin, Colistin, fosfomycin, erythromycin, azithromycin, spiramycin, telithromycin, ciprofloxacin, sulfamethoxazole, doxycycline, tetracycline, minocycline, and trimethoprim. | AslA, air, anr, cea, chuA, cia, cib, csgA, eilA, etsC, fdeC, fimH, gad, hha, hlyE, hlyF, hra, iha, iss, iucC, iutA, kpsE, kpsMII, nlpI, ompT, papA_F19, papC, shiB, sitA, terC, traJ, traT, yehA, yehB, yehC, yehD |
| **AAS-18** |  | ST10 | 92.2% | O41:H45 | fumC7, fimH23 | aph(3')-Ia, mcr-1.1, fosA4, mph(A), qnrS1, qnrB19, tet(B), tet(M), dfrA14 | Kanamycin, an unknown aminoglycoside, Neomycin, Lividomycin, Paromomycin, Ribostamycin, Colistin, Fosfomycin, Erythromycin, Azithromycin, Spiramycin, Telithromycin, Ciprofloxacin, Doxycycline, Tetracycline, Minocycline, and Trimethoprim. | AslA, anr, cia, cma, csgA, cvaC, fdeC, fimH, gad, hlyE, hlyF, iroN, iss, iucC, iutA, nlpI, ompT, sitA, terC, traJ, traT, yehA, yehB, yehC, yehD |
| **AAS-21** |  | ST3489 | 92% | O86:H32 | fumC11, fimH54 | aac(3)-VIa, aph(6)-Id, aph(3'')-Ib, aph(3')-Ia, aadA1, mcr-1.1, mph(A), floR, sul1, sul2, tet(A), tet(X4), dfrA12 | Gentamicin, Sisomicin, Netilmicin, Tobramycin, Streptomycin, Kanamycin, Neomycin, an unknown aminoglycoside, Lividomycin, Paromomycin, Ribostamycin, Spectinomycin, Colistin, Erythromycin, Azithromycin, Spiramycin, Telithromycin, Chloramphenicol, Florfenicol, Sulfamethoxazole, Doxycycline, Tetracycline, Minocycline, Tigecycline, and Trimethoprim. | AslA, anr, cea, cma, csgA, fdeC, fimH, gad, hlyE, hra, Iha, IreA, Iss, IucC, IutA, NlpI, ShiB, SitA, TerC, traJ, traT, yehA, yehB, yehC, yehD |

**Table S3. Mobile genetic elements of wild-type strain K12**.


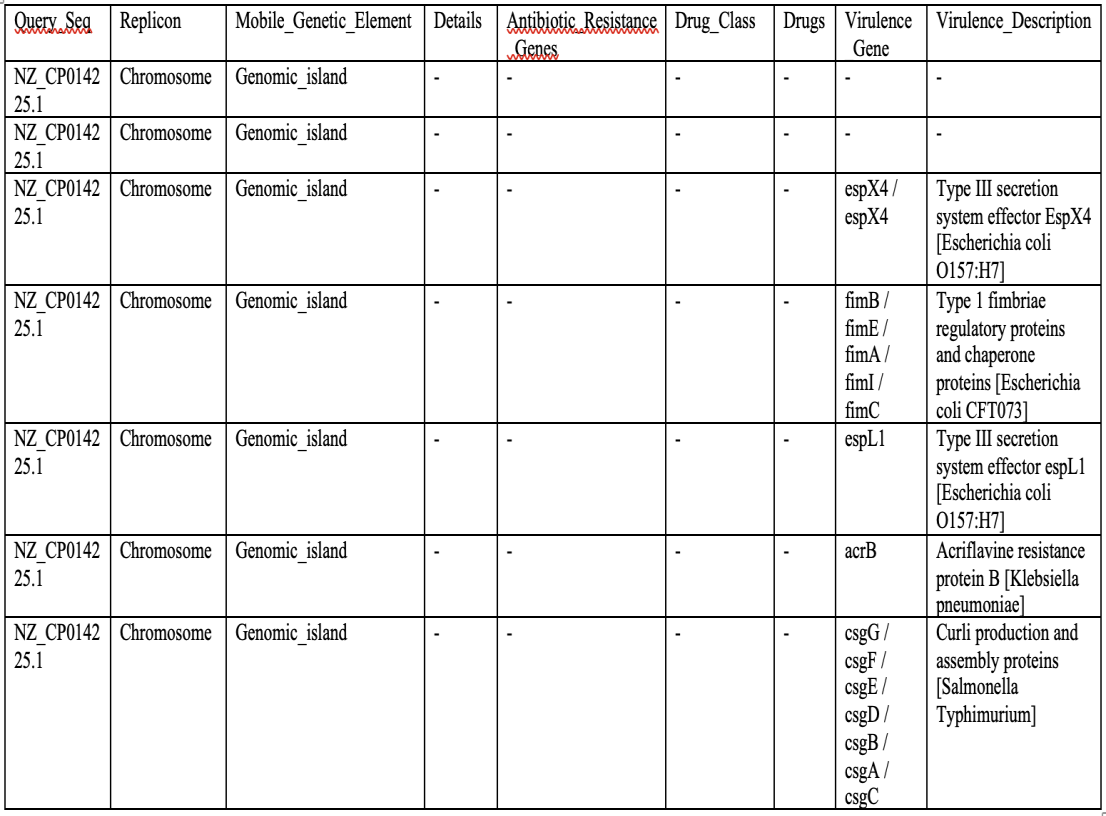


**Table S4. Mobile genetic elements of strain AAS-14.**


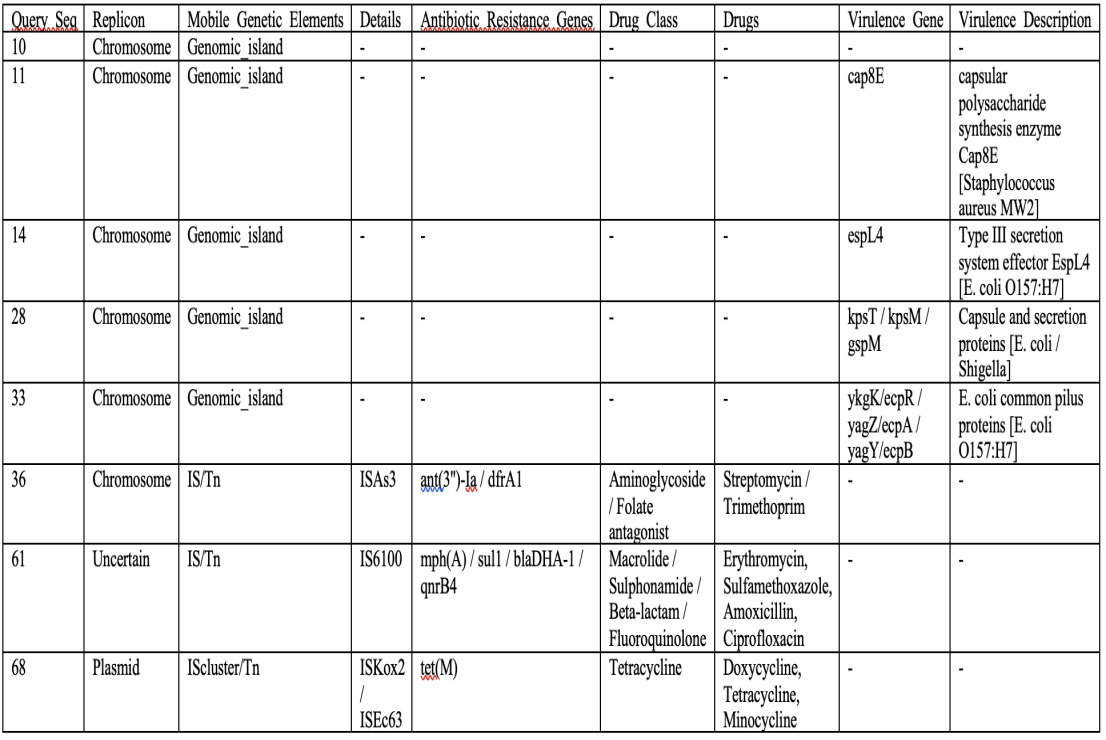


**Table S5. Mobile genetic elements of strain AAS-18**.


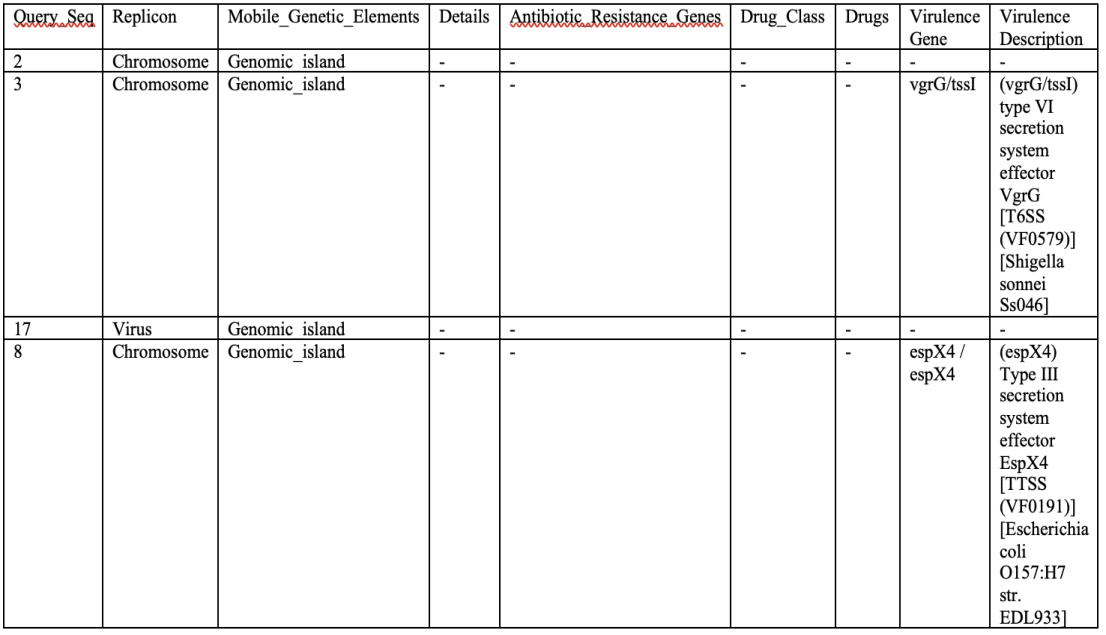


**Table S6. Mobile genetic elements of strain 21**.


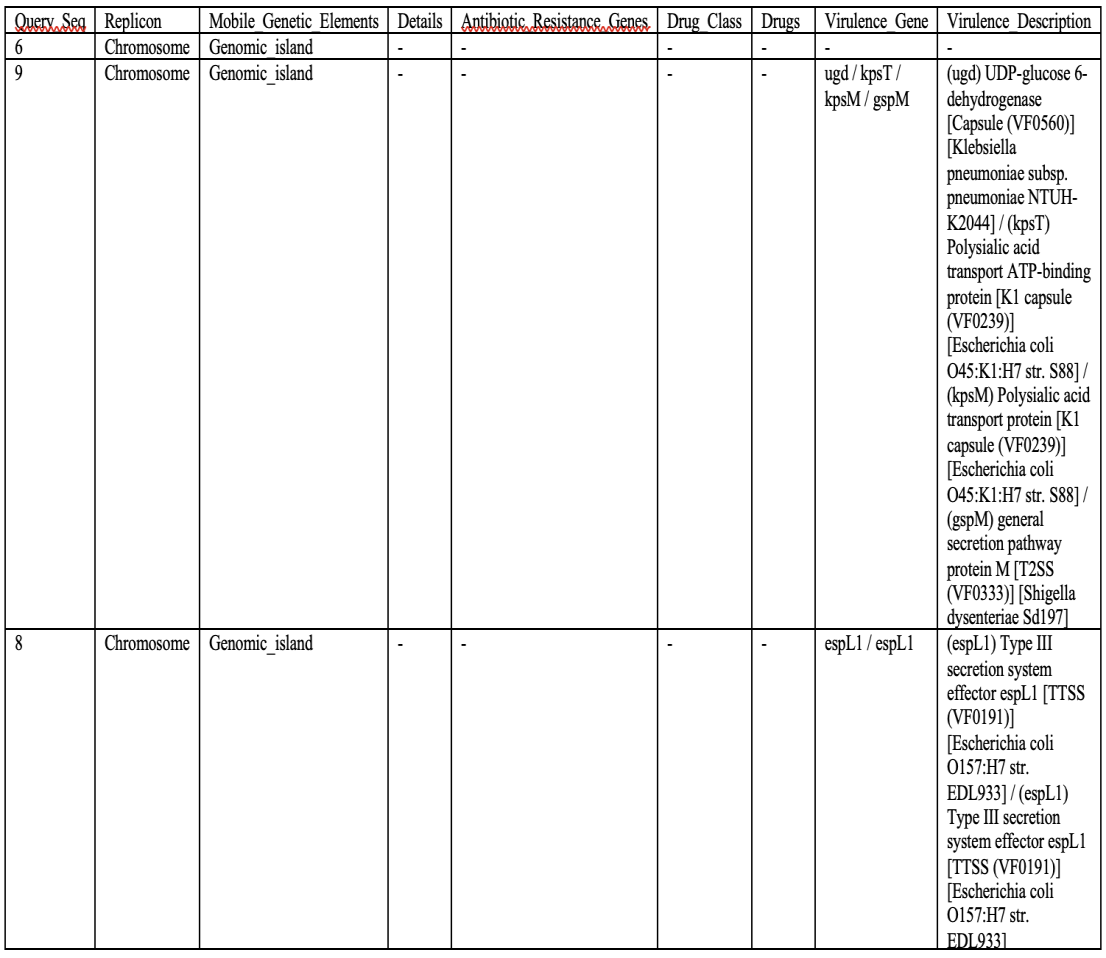


**Table S7. Virulence genes carried by the *E. coli* strains and the encoded function**.

| **Gene** | **Function** | **Reference** |
| --- | --- | --- |
| AslA (Argininosuccinate lyase) | Adhesion | [1] |
| fimH (Fimbrial adhesin H) | Adhesion | [2] |
| gad (Glutamate decarboxylase) | Resistance of acids as defense mechanism | [3] |
| ompT (Outer membrane protease T) | Degrades proteins that have antimicrobial function | [4] |
| terC (Tellurite resistance C) | Resistance conferral to tellurite metal | [5] |
| yehA (Putative fimbrial protein YehA) | Adhesion | [6] |
| hlyE (Hemolysin E) | Host cell lysis | [7] |
| iss (Increased serum survival) | Evade host immune system | [8] |
| nlpI (New lipoprotein I) | Vesicle formation | [9] |
| yehB (Putative fimbrial protein YehB) | Structure of fibmbria | [6] |
| yehC (Fimbrial chaperone YehC) | Structure of fibmbria | [6] |
| yehD (Fimbrial usher YehD) | Structure of fibmbria | [6] |
| iutA (Iron uptake transporter A) | Iron receptor | [10] |
| kpsE (Capsule synthesis E) | Capsule synthesis | [11] |
| kpsMII (Capsule export M II) | Capsule structure | [12] |
| eilA (Enteric invasin-like A) | Transcriptional regulator of genes associated with virulence | [13] |
| etsC (Enterotoxin secretion C) | Protein secretion | [14] |
| fdeC (Fimbrial determinant E C) | Adhesion | [15] |
| hlyF (Hemolysin F) | Virulence lysing protein | [14] |
| air (Adhesin involved in colonization) | Adhesion | [16] |
| anr (Anaerobic regulator) | Expression regulation | [17] |
| cea (Colicin E activity) | Bacteriocin | [18] |
| traT (Transfer outer membrane protein T) | Serum resistance | [14] |
| cma (Colicin M activity) | Bacteriocin | [19] |
| cvaC (Colicin V activity C) | Colicin V | [14] |
| hra (Heat-resistant agglutinin) | Adhesion | [20] |
| iha (Iron-regulated gene A homolog adhesin) | Adhesion | [21] |
| iucC (Iron uptake component C) | Iron uptake | [14] |
| ompP (Outer membrane protein P) | Membrane stability | [4] |
| papA_F19 (P fimbrial structural subunit A F19) | Fimbrial structure | [22] |
| papC (P fimbrial usher protein C) | Fimbrial structure | [16] |
| shiB (Shiga toxin immunity protein B) | Colicin protection | [23] |
| sitA (Siderophore iron transport A) | Iron, manganese transport | [14] |
| traJ (Transfer operon regulator J) | Plasmid transfer regulation | [23] |
| cia (Colicin I activity) | Invasion | [23] |
| cib (Colicin B) | Bacteriocin targeting others | [23] |
| iroN (Iron-regulated outer membrane receptor N) | Receptor of salmochelin siderophore (iron) | [14] |

**References for supplementary data**

1. Aguirre-Sanchez, J.R., et al., *Phylogenetic group and virulence profile classification in Escherichia coli from distinct isolation sources in Mexico.* Infect Genet Evol, 2022. **106**: p. 105380.

2. Foroogh, N., et al., *Structural and functional characterization of the FimH adhesin of uropathogenic Escherichia coli and its novel applications.* Microb Pathog, 2021. **161**(Pt B): p. 105288.

3. Moreau, P.L., *The lysine decarboxylase CadA protects Escherichia coli starved of phosphate against fermentation acids.* J Bacteriol, 2007. **189**(6): p. 2249-61.

4. Kukkonen, M. and T.K. Korhonen, *The ompt in family of enterobacterial surface proteases/adhesins: from housekeeping in Escherichia coli to systemic spread of Yersinia pestis.* Int J Med Microbiol, 2004. **294**(1): p. 7-14.

5. Vavrova, S., et al., *The tellurite resistance gene cluster of pathogenic bacteria and its effect on oxidative stress response.* Folia Microbiol (Praha), 2024. **69**(2): p. 433-444.

6. Gonyar, L.A., et al., *The yad and yeh fimbrial loci influence gene expression and virulence in enterohemorrhagic Escherichia coli O157:H7.* mSphere, 2024. **9**(7): p. e0012424.

7. Hunt, S., J. Green, and P.J. Artymiuk, *Hemolysin E (HlyE, ClyA, SheA) and related toxins.* Adv Exp Med Biol, 2010. **677**: p. 116-26.

8. Biran, D., et al., *Surviving Serum: the Escherichia coli iss Gene of Extraintestinal Pathogenic E. coli Is Required for the Synthesis of Group 4 Capsule.* Infect Immun, 2021. **89**(10): p. e0031621.

9. Mathelie-Guinlet, M., et al., *Stress-Induced Catch-Bonds to Enhance Bacterial Adhesion.* Trends Microbiol, 2021. **29**(4): p. 286-288.

10. Ikeda, G., et al., *Mitochondria-Rich Extracellular Vesicles From Autologous Stem Cell-Derived Cardiomyocytes Restore Energetics of Ischemic Myocardium.* J Am Coll Cardiol, 2021. **77**(8): p. 1073-1088.

11. Rosenow, C., I.S. Roberts, and K. Jann, *Isolation from recombinant Escherichia coli and characterization of CMP-Kdo synthetase, involved in the expression of the capsular K5 polysaccharide (K-CKS).* FEMS Microbiol Lett, 1995. **125**(2-3): p. 159-64.

12. Karbalaei, M., et al., *Comprehensive Analysis of blaCTX-M1 Gene Expression Alongside iutA, csgA, and kpsMII Virulence Genes in Septicemic Escherichia coli Using Real-Time PCR.* Microorganisms, 2025. **13**(1).

13. Petro, C.D., et al., *Genetic and Virulence Profiles of Enteroaggregative Escherichia coli (EAEC) Isolated From Deployed Military Personnel (DMP) With Travelers' Diarrhea.* Front Cell Infect Microbiol, 2020. **10**: p. 200.

14. Hammad, A.M., et al., *Pathogenome comparison and global phylogeny of Escherichia coli ST1485 strains.* Sci Rep, 2022. **12**(1): p. 18495.

15. Aleksandrowicz, A., et al., *FdeC expression regulates motility and adhesion of the avian pathogenic Escherichia coli strain IMT5155.* Vet Res, 2024. **55**(1): p. 70.

16. Bujnakova, D., L. Karahutova, and V. Kmet, *Escherichia coli Specific Virulence-Gene Markers Analysis for Quality Control of Ovine Cheese in Slovakia.* Microorganisms, 2021. **9**(9).

17. Rodriguez-Valverde, D., et al., *Highly-conserved regulatory activity of the ANR family in the virulence of diarrheagenic bacteria through interaction with master and global regulators.* Sci Rep, 2023. **13**(1): p. 7024.

18. Muenzner, J., et al., *Cellular and viral peptides bind multiple sites on the N-terminal domain of clathrin.* Traffic, 2017. **18**(1): p. 44-57.

19. Yang, S.C., et al., *Current pathogenic Escherichia coli foodborne outbreak cases and therapy development.* Arch Microbiol, 2017. **199**(6): p. 811-825.

20. Srinivasan, K., et al., *Netrin-1/neogenin interaction stabilizes multipotent progenitor cap cells during mammary gland morphogenesis.* Dev Cell, 2003. **4**(3): p. 371-82.

21. Colello, R., et al., *Expression of hes, iha, and tpsA codified in locus of adhesion and autoaggregation and their involvement in the capability of shiga toxin-producing Escherichia coli strains to adhere to epithelial cells.* BMC Res Notes, 2023. **16**(1): p. 163.

22. Li, H., et al., *Prevalence of Escherichia coli and Antibiotic Resistance in Animal-Derived Food Samples - Six Districts, Beijing, China, 2020.* China CDC Wkly, 2021. **3**(47): p. 999-1004.

23. Awosile, B., et al., *Genomic Characterization of Fecal Escherichia coli Isolates with Reduced Susceptibility to Beta-Lactam Antimicrobials from Wild Hogs and Coyotes.* Pathogens, 2023. **12**(7).
