# Supplementary material for: Biochemical and genomic characterization of last-resort antibiotic resistance and oxidant tolerance in environmental E. coli
Source: Front Toxicol. 2026 Jun 3;8:1822843. doi: 10.3389/ftox.2026.1822843 (PMC13271735; doi:10.3389/ftox.2026.1822843)
Supplement: Supplementary file 4 [file DataSheet1.pdf]

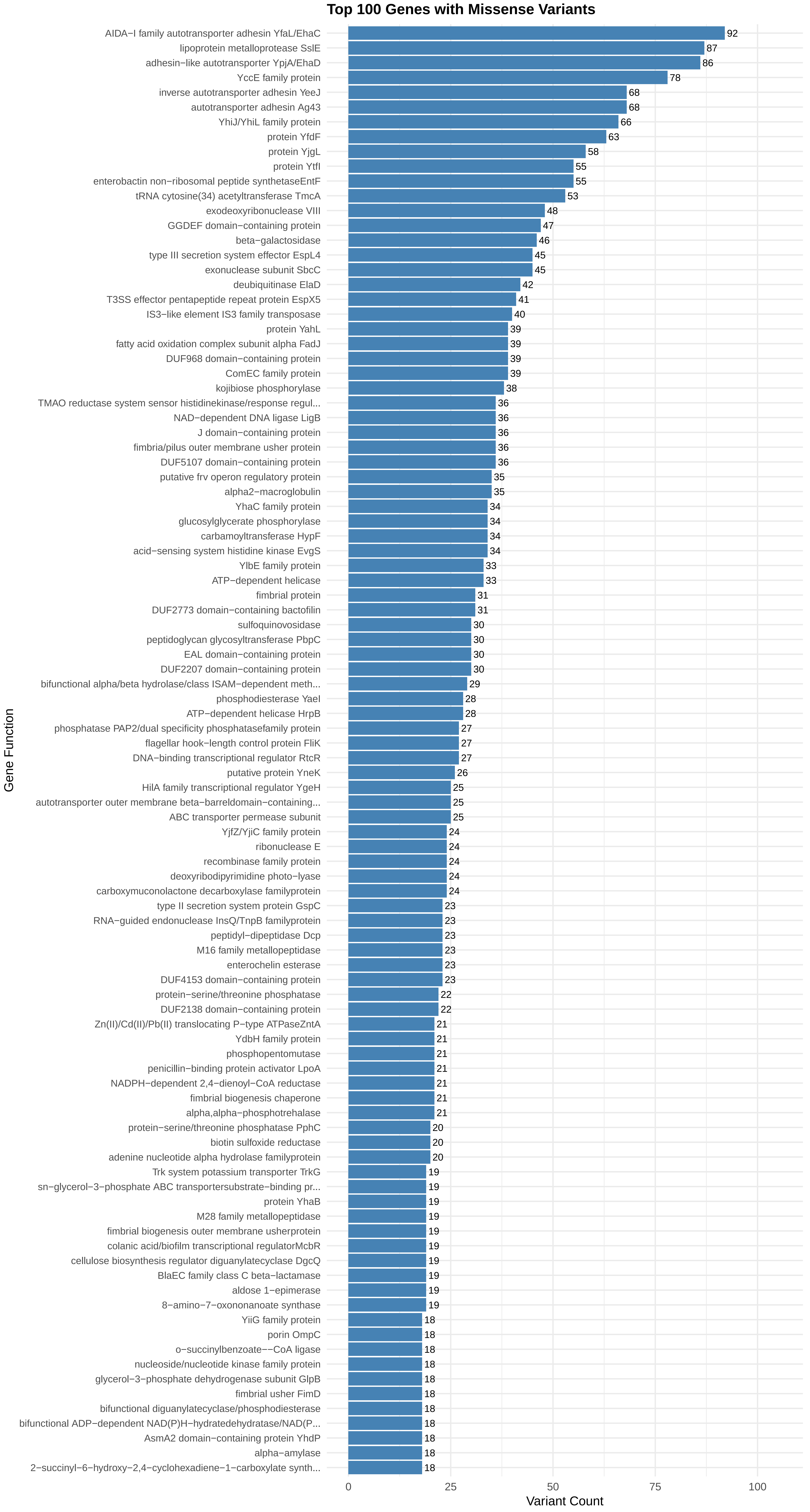

Top 100 Genes with Missense Variants
AIDA−I family autotransporter adhesin YfaL/EhaC 92
lipoprotein metalloprotease SslE 87
adhesin−like autotransporter YpjA/EhaD 86
YccE family protein 78
inverse autotransporter adhesin YeeJ 68
autotransporter adhesin Ag43 68
YhiJ/YhiL family protein 66
protein YfdF 63
protein YjgL 58
protein YtfI 55
enterobactin non−ribosomal peptide synthetaseEntF 55
tRNA cytosine(34) acetyltransferase TmcA 53
exodeoxyribonuclease VIII 48
GGDEF domain−containing protein 47
beta−galactosidase 46
type III secretion system effector EspL4 45
exonuclease subunit SbcC 45
deubiquitinase ElaD 42
T3SS effector pentapeptide repeat protein EspX5 41
IS3−like element IS3 family transposase 40
protein YahL 39
fatty acid oxidation complex subunit alpha FadJ 39
DUF968 domain−containing protein 39
ComEC family protein 39
kojibiose phosphorylase 38
TMAO reductase system sensor histidinekinase/response regul... 36
NAD−dependent DNA ligase LigB 36
J domain−containing protein 36
fimbria/pilus outer membrane usher protein 36
DUF5107 domain−containing protein 36
putative frv operon regulatory protein 35
alpha2−macroglobulin 35
YhaC family protein 34
glucosylglycerate phosphorylase 34
carbamoyltransferase HypF 34
acid−sensing system histidine kinase EvgS 34
YlbE family protein 33
ATP−dependent helicase 33
fimbrial protein 31
DUF2773 domain−containing bactofilin 31
sulfoquinovosidase 30
peptidoglycan glycosyltransferase PbpC 30
EAL domain−containing protein 30
DUF2207 domain−containing protein 30
bifunctional alpha/beta hydrolase/class ISAM−dependent meth... 29
phosphodiesterase YaeI 28
ATP−dependent helicase HrpB 28
phosphatase PAP2/dual specificity phosphatasefamily protein 27
flagellar hook−length control protein FliK 27
DNA−binding transcriptional regulator RtcR 27
putative protein YneK 26
HilA family transcriptional regulator YgeH 25
autotransporter outer membrane beta−barreldomain−containing... 25
ABC transporter permease subunit 25
YjfZ/YjiC family protein 24
ribonuclease E 24
recombinase family protein 24
deoxyribodipyrimidine photo−lyase 24
carboxymuconolactone decarboxylase familyprotein 24
type II secretion system protein GspC 23
RNA−guided endonuclease InsQ/TnpB familyprotein 23
peptidyl−dipeptidase Dcp 23
M16 family metallopeptidase 23
enterochelin esterase 23
DUF4153 domain−containing protein 23
protein−serine/threonine phosphatase 22
DUF2138 domain−containing protein 22
Zn(II)/Cd(II)/Pb(II) translocating P−type ATPaseZntA 21
YdbH family protein 21
phosphopentomutase 21
penicillin−binding protein activator LpoA 21
NADPH−dependent 2,4−dienoyl−CoA reductase 21
fimbrial biogenesis chaperone 21
alpha,alpha−phosphotrehalase 21
protein−serine/threonine phosphatase PphC 20
biotin sulfoxide reductase 20
adenine nucleotide alpha hydrolase familyprotein 20
Trk system potassium transporter TrkG 19
sn−glycerol−3−phosphate ABC transportersubstrate−binding pr... 19
protein YhaB 19
M28 family metallopeptidase 19
fimbrial biogenesis outer membrane usherprotein 19
colanic acid/biofilm transcriptional regulatorMcbR 19
cellulose biosynthesis regulator diguanylatecyclase DgcQ 19
BlaEC family class C beta−lactamase 19
aldose 1−epimerase 19
8−amino−7−oxononanoate synthase 19
YiiG family protein 18
porin OmpC 18
o−succinylbenzoate−−CoA ligase 18
nucleoside/nucleotide kinase family protein 18
glycerol−3−phosphate dehydrogenase subunit GlpB 18
fimbrial usher FimD 18
bifunctional diguanylatecyclase/phosphodiesterase 18
bifunctional ADP−dependent NAD(P)H−hydratedehydratase/NAD(P... 18
AsmA2 domain−containing protein YhdP 18
alpha−amylase 18
2−succinyl−6−hydroxy−2,4−cyclohexadiene−1−carboxylate synth... 18
Gene Function
0
25
50
75
100
Variant Count

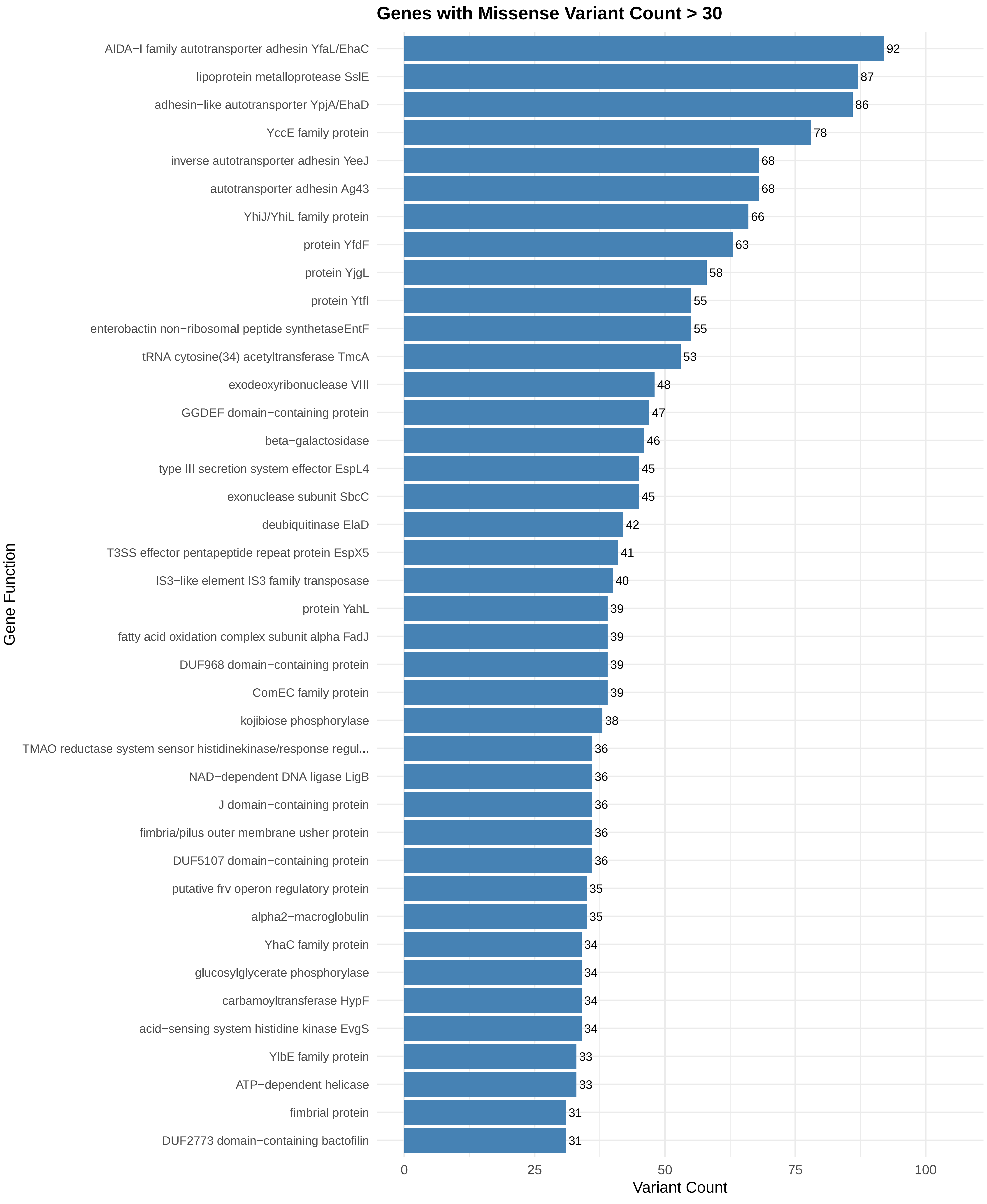

Genes with Missense Variant Count > 30
AIDA−I family autotransporter adhesin YfaL/EhaC
92
lipoprotein metalloprotease SslE
87
adhesin−like autotransporter YpjA/EhaD
86
YccE family protein
78
inverse autotransporter adhesin YeeJ
68
autotransporter adhesin Ag43
68
YhiJ/YhiL family protein
66
protein YfdF
63
protein YjgL
58
protein YtfI
55
enterobactin non−ribosomal peptide synthetaseEntF
55
tRNA cytosine(34) acetyltransferase TmcA
53
exodeoxyribonuclease VIII
48
GGDEF domain−containing protein
47
beta−galactosidase
46
type III secretion system effector EspL4
45
exonuclease subunit SbcC
45
deubiquitinase ElaD
42
T3SS effector pentapeptide repeat protein EspX5
41
IS3−like element IS3 family transposase
40
protein YahL
39
fatty acid oxidation complex subunit alpha FadJ
39
DUF968 domain−containing protein
39
ComEC family protein
39
kojibiose phosphorylase
38
TMAO reductase system sensor histidinekinase/response regul...
36
NAD−dependent DNA ligase LigB
36
J domain−containing protein
36
fimbria/pilus outer membrane usher protein
36
DUF5107 domain−containing protein
36
putative frv operon regulatory protein
35
alpha2−macroglobulin
35
YhaC family protein
34
glucosylglycerate phosphorylase
34
carbamoyltransferase HypF
34
acid−sensing system histidine kinase EvgS
34
YlbE family protein
33
ATP−dependent helicase
33
fimbrial protein
31
DUF2773 domain−containing bactofilin
31
Gene Function
0
25
50
75
100
Variant Count

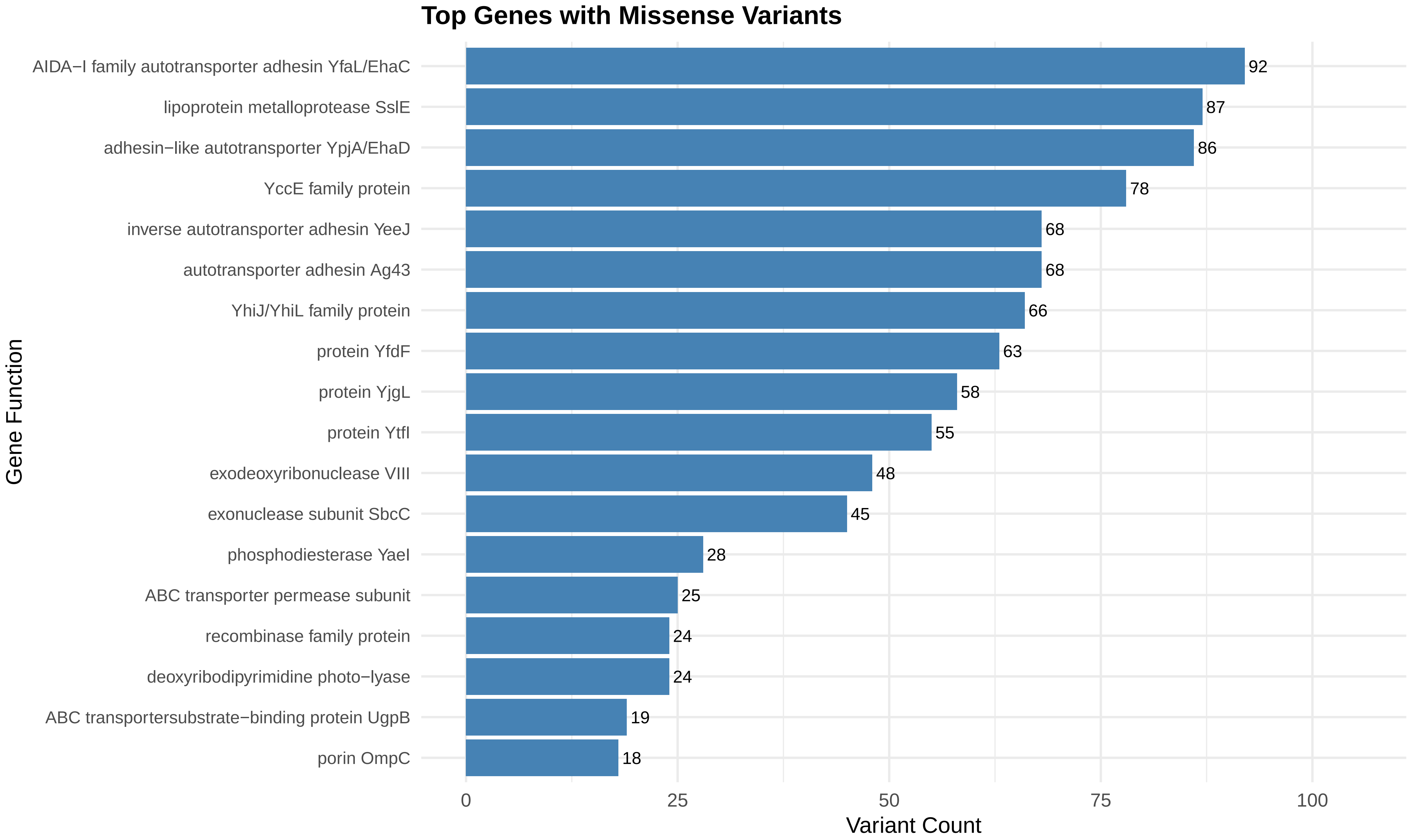
Top Genes with Missense Variants
AIDA−I family autotransporter adhesin YfaL/EhaC
lipoprotein metalloprotease SslE
adhesin−like autotransporter YpjA/EhaD
YccE family protein
inverse autotransporter adhesin YeeJ
autotransporter adhesin Ag43
YhiJ/YhiL family protein
protein YfdF
protein YjgL
protein YtfI
exodeoxyribonuclease VIII
exonuclease subunit SbcC
phosphodiesterase YaeI
ABC transporter permease subunit
recombinase family protein
deoxyribodipyrimidine photo−lyase
ABC transportersubstrate−binding protein UgpB
porin OmpC
92
87
86
78
68
68
66
63
58
55
48
45
28
25
24
24
19
18
Gene Function
0
25
50
75
100
Variant Count

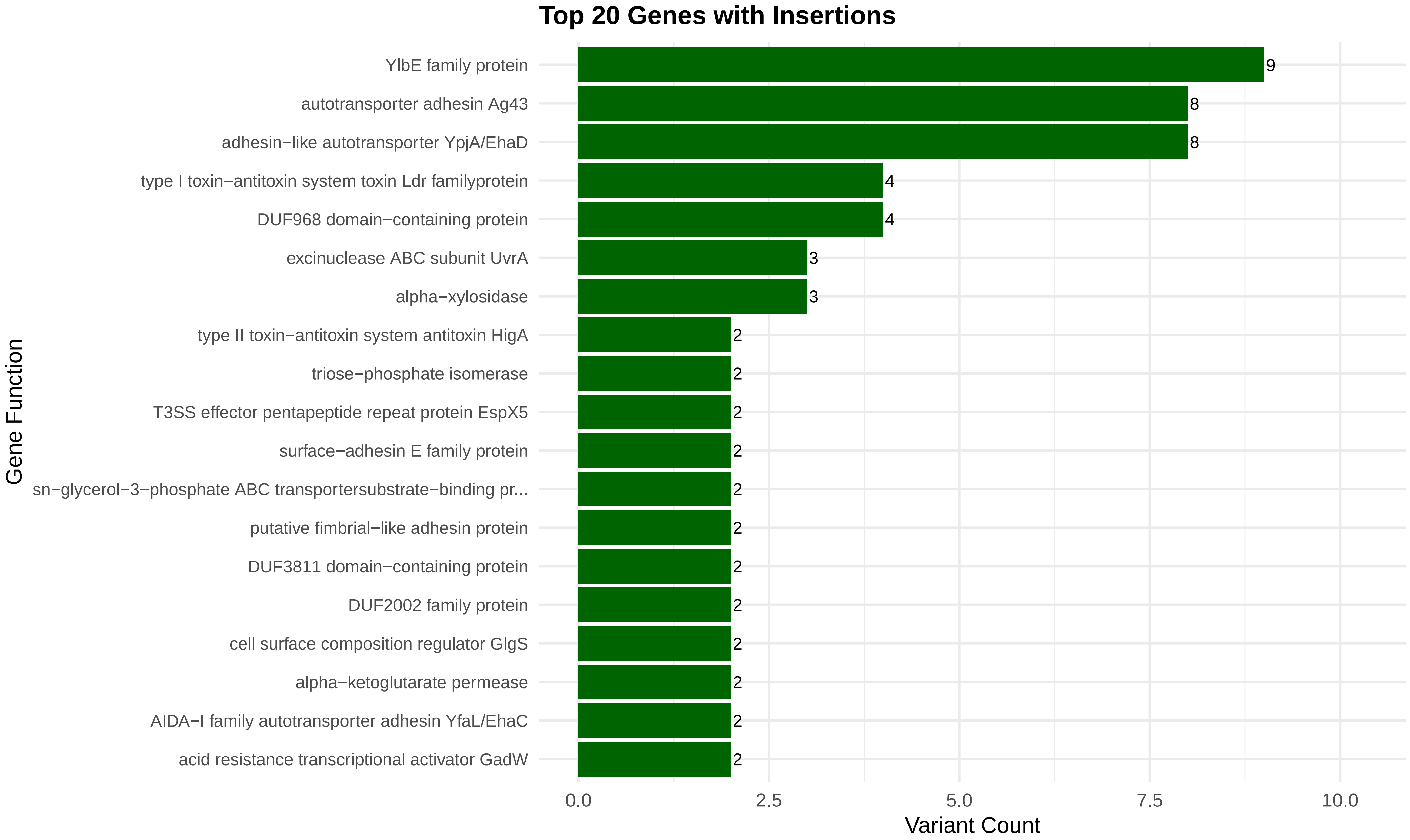
Top 20 Genes with Insertions
Gene Function
Variant Count
YlbE family protein
autotransporter adhesin Ag43
adhesin−like autotransporter YpjA/EhaD
type I toxin−antitoxin system toxin Ldr familyprotein
DUF968 domain−containing protein
excinuclease ABC subunit UvrA
alpha−xylosidase
type II toxin−antitoxin system antitoxin HigA
triose−phosphate isomerase
T3SS effector pentapeptide repeat protein EspX5
surface−adhesin E family protein
sn−glycerol−3−phosphate ABC transportersubstrate−binding pr...
putative fimbrial−like adhesin protein
DUF3811 domain−containing protein
DUF2002 family protein
cell surface composition regulator GlgS
alpha−ketoglutarate permease
AIDA−I family autotransporter adhesin YfaL/EhaC
acid resistance transcriptional activator GadW
9
8
8
4
4
3
3
2
2
2
2
2
2
2
2
2
2
2
2
0.0
2.5
5.0
7.5
10.0

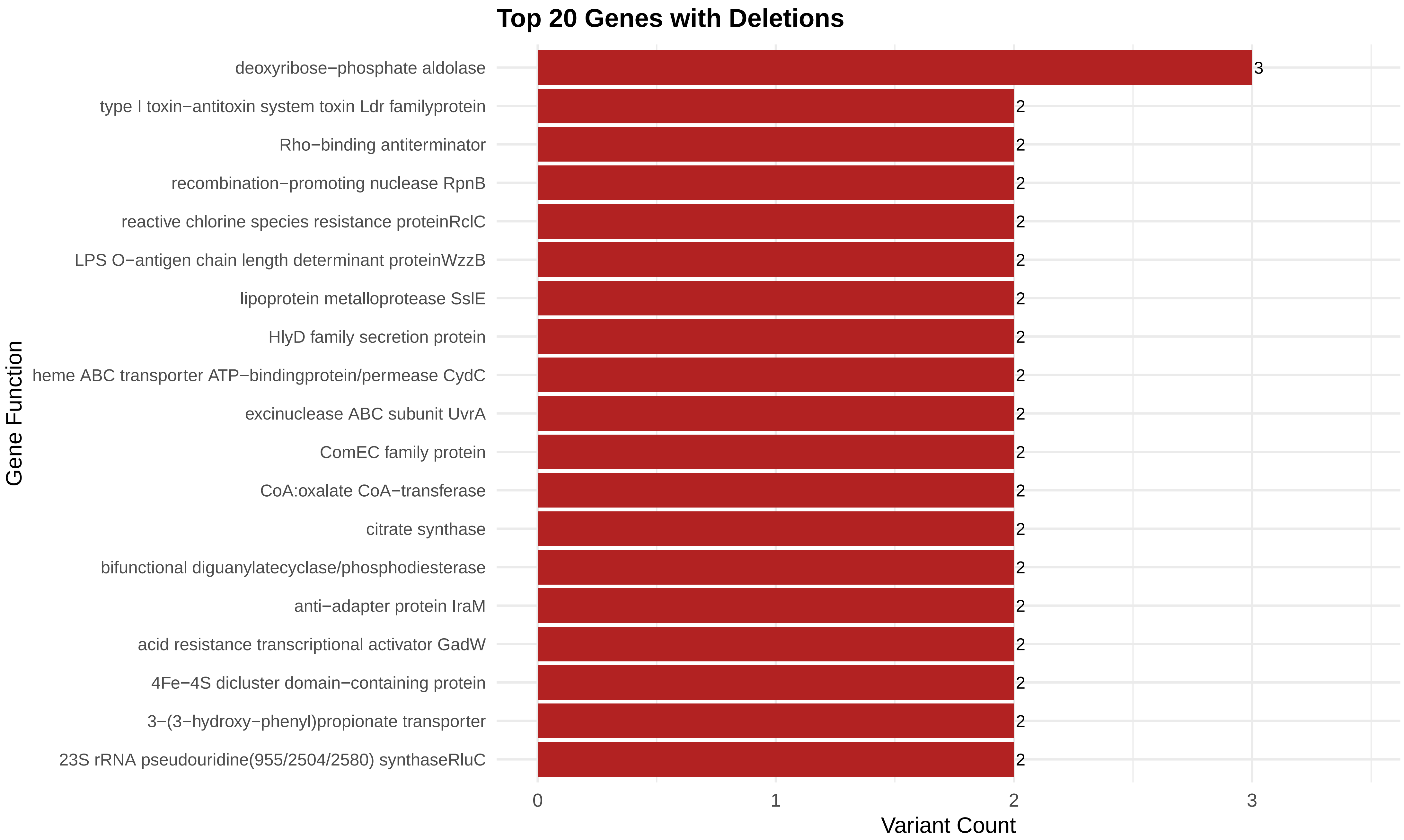

Top 20 Genes with Deletions
deoxyribose−phosphate aldolase
type I toxin−antitoxin system toxin Ldr familyprotein
Rho−binding antiterminator
recombination−promoting nuclease RpnB
reactive chlorine species resistance proteinRclC
LPS O−antigen chain length determinant proteinWzzB
lipoprotein metalloprotease SslE
HlyD family secretion protein
heme ABC transporter ATP−bindingprotein/permease CydC
excinuclease ABC subunit UvrA
ComEC family protein
CoA:oxalate CoA−transferase
citrate synthase
bifunctional diguanylatecyclase/phosphodiesterase
anti−adapter protein IraM
acid resistance transcriptional activator GadW
4Fe−4S dicluster domain−containing protein
3−(3−hydroxy−phenyl)propionate transporter
23S rRNA pseudouridine(955/2504/2580) synthaseRluC
3
2
2
2
2
2
2
2
2
2
2
2
2
2
2
2
2
2
2
0
1
2
3
Variant Count
Gene Function
